# Microcavity- and Microlaser-Based Optical Barcoding: A Review of Encoding Techniques and Applications

**DOI:** 10.1021/acsphotonics.2c01611

**Published:** 2023-05-02

**Authors:** Abdur Rehman Anwar, Maruša Mur, Matjaž Humar

**Affiliations:** † Department of Condensed Matter Physics, J. Stefan Institute, Jamova 39, SI-1000 Ljubljana, Slovenia; ‡ CENN Nanocenter, Jamova 39, SI-1000 Ljubljana, Slovenia; § Faculty of Mathematics and Physics, University of Ljubljana, Jadranska 19, SI-1000 Ljubljana, Slovenia

**Keywords:** barcodes, tagging, microcavities, microlasers, cell tracking

## Abstract

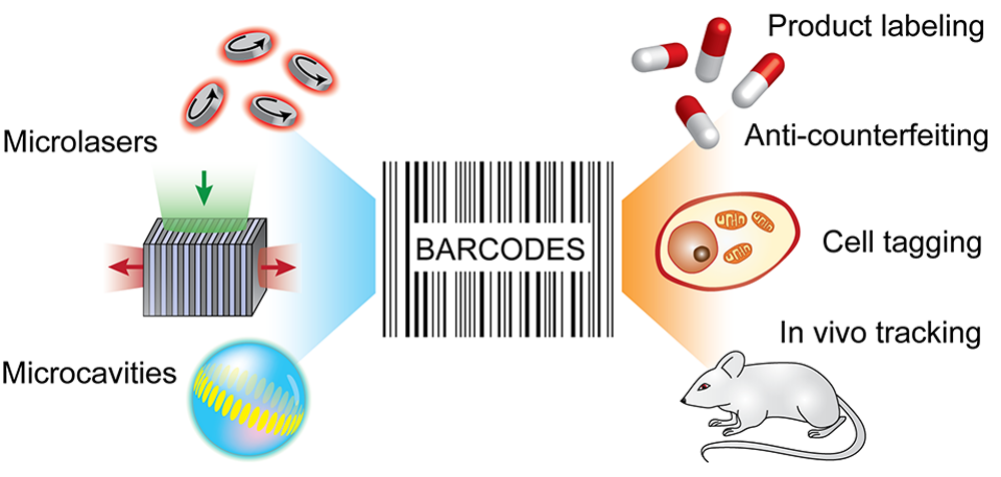

Optical microbarcodes have recently received a great deal of interest because of their suitability for a wide range of applications, such as multiplexed assays, cell tagging and tracking, anticounterfeiting, and product labeling. Spectral barcodes are especially promising because they are robust and have a simple readout. In addition, microcavity- and microlaser-based barcodes have very narrow spectra and therefore have the potential to generate millions of unique barcodes. This review begins with a discussion of the different types of barcodes and then focuses specifically on microcavity-based barcodes. While almost any kind of optical microcavity can be used for barcoding, currently whispering-gallery microcavities (in the form of spheres and disks), nanowire lasers, Fabry–Pérot lasers, random lasers, and distributed feedback lasers are the most frequently employed for this purpose. In microcavity-based barcodes, the information is encoded in various ways in the properties of the emitted light, most frequently in the spectrum. The barcode is dependent on the properties of the microcavity, such as the size, shape, and the gain materials. Various applications of these barcodes, including cell tracking, anticounterfeiting, and product labeling are described. Finally, the future prospects for microcavity- and microlaser-based barcodes are discussed.

## Introduction

Traditional linear or one-dimensional barcodes are made up of parallel vertical lines or bars of different widths and spacings in between, which led to the use of the term *barcode*. In our daily lives, these barcodes are extensively used to label macroscopic items such as products in shops and postal packages, as well as for healthcare and industrial applications by providing the rapid, accurate, and simple identification of items.^1,2^ Microbarcodes serve the same purpose as macroscopic barcodes, i.e., tagging an object so as to be able to identify it later, but at a much smaller scale. They have attracted a great deal of attention because of their potential in tracking, labeling, biodetection, cell tagging, information security, and anticounterfeiting.^3−5^ This is especially so in the biosciences, where the high-throughput screening and multiplexed assaying of samples and heterogeneous cell populations have driven the investigation of novel barcoding techniques at the micro- and nanoscales.

The real-time tracking of large numbers of cells is extremely important for understanding biological activity, e.g., for observing the response of different types of cells in complex environments, such as cancer metastasis, cell heterogeneity, cell differentiation and cell therapy.^6,7^ In simple experiments cells can be tracked using regular microscopy. However, in more complex cases with a large number of motile cells and for in vivo experiments this is not practical. The tagging of cells with unique barcodes makes it possible to track cellular identities in space and time or over the course of long or multiple measurements for different time periods. This enables discoveries of cellular behaviors that are otherwise masked by averaging over large ensembles.^8,9^ Usually, different cell populations are tagged with fluorescent labels. The combinatorial expression of multiple fluorescent proteins such as Brainbow, which can generate up to 100 colors by ratiometric coding,^10−12^ is especially prominent. Fluorescent tagging is, however, prone to errors with thick tissues and due to the effect of photobleaching. Other currently used methods for cell tagging and tracking include those using nanoparticles,^13^ quantum dots,^14,15^ DNA (or RNA),^16−18^ lifetime tagging with lanthanide particles,^19,20^ Raman probes,^21,22^ etc.

Microbarcodes have also been used for information security and anticounterfeiting. In our modern, information-intensive society anticounterfeiting plays a key role in information storage, identity recognition, and document encryption. Optical anticounterfeiting, including photonic barcodes with a spectral signature, have gained a lot of attention for anticounterfeiting applications due to the easy readout and the high storage capacity.^23^

**Figure 1:**
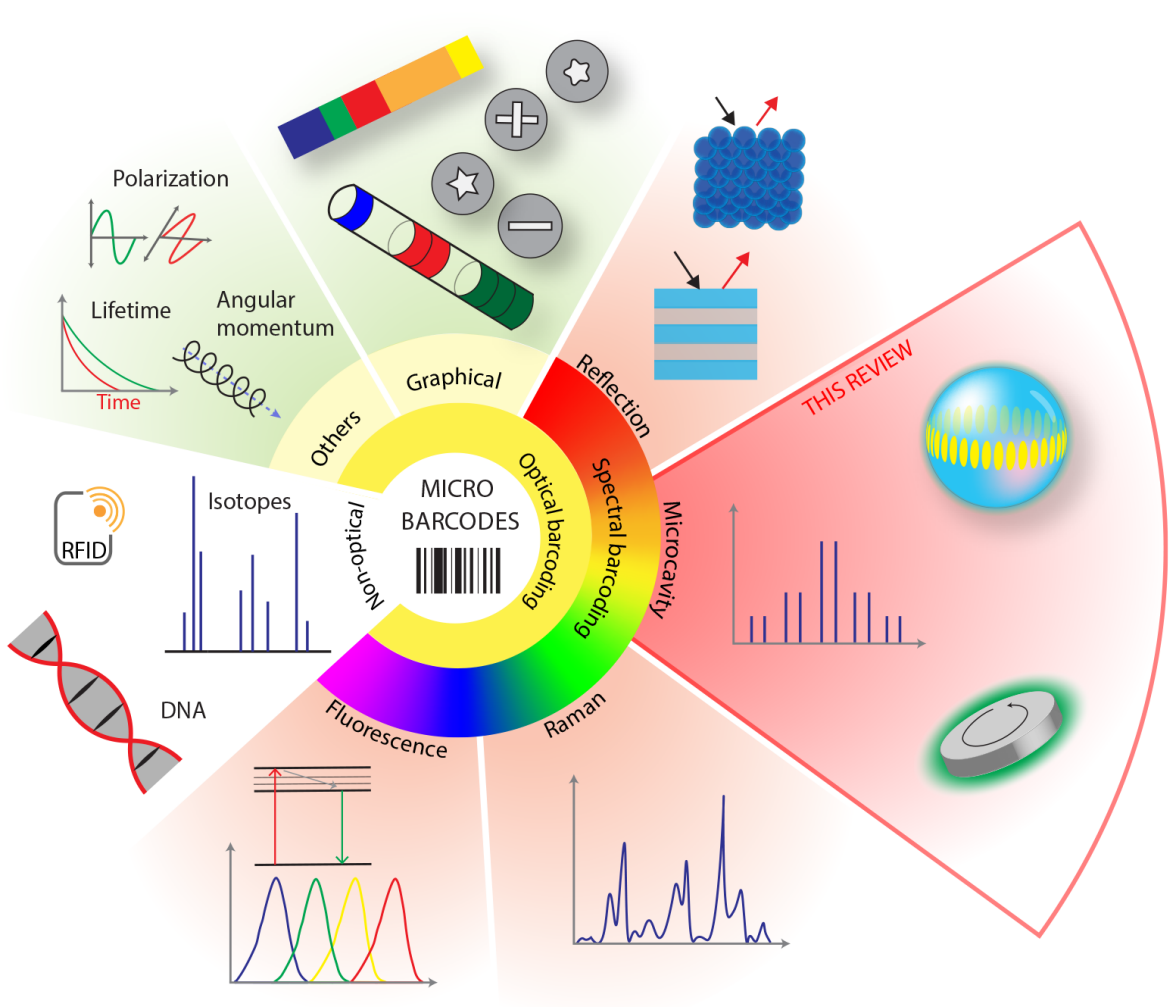
Categorization of different types of microbarcodes. Based on the readout, they are divided into optical and nonoptical. Optical barcodes encode the information via different properties, such as shape, color, light spectrum, polarization and angular momentum, and lifetime. Spectral barcodes are mainly based on fluorescence, Raman scattering, photonic crystals, and microcavities. In this review we focus specifically on the microcavity barcodes, which most frequently encode the information via the spectrum.

Barcodes can store information in a very wide variety of ways, which also determines the way a barcode is read (Figure 1). Frequently, microbarcodes are read optically, but there are a number of nonoptical barcoding schemes. Examples include radio frequency identification (RFID),^24^ DNA (or RNA),^16−18^ and isotope barcoding.^25^ While DNA and isotope barcoding are very powerful techniques, the readout is relatively slow and complex compared to optical barcodes. These techniques are also damaging to the object, since a small sample has to be collected for analysis.

The term barcoding is sometimes also used for barcodes that are already naturally present in an object. One obvious example is DNA, which is unique for each individual organism. The other example is isotopic fingerprinting, where the geographical origin of food products is determined.^26^ Conversely, all the barcodes discussed in this review are artificial and have to be added to the object that we want to label.

In optical barcoding, the optical properties of the encoding element are utilized, e.g., a graphical pattern, fluorescence spectra, and light-scattering properties.^27^ The greatest advantage of optical barcodes is the ability to read them remotely, either passively or actively. A passive readout uses only the ambient light and a detector. An example would be reading a QR (quick response) code with a camera on a smartphone. The microcavity-based barcodes reviewed here all need to be excited actively by an artificial light source, typically at a shorter wavelength, while the fluorescence is detected at a longer wavelength. Further, the microlasers need to be pumped above their laser threshold with a pulsed laser to generate the signal. An active readout is especially useful in anticounterfeiting applications where a barcode can be made in such a way that it can only give the right information if actively read in a particular way, for example, by illuminating it with the correct wavelength or intensity. The readout of optical barcodes is also nondestructive and practically instantaneous, frequently well below one second. Furthermore, optical barcodes provide a large encoding capacity and can be made with a microscopic size—even a single molecule can be used for this purpose. Optical microbarcodes are typically in the range 1–1000 μm. Their small size is their biggest advantage compared to regular barcodes such as QR (typically centimeter or larger in size) and RFID (millimeter to centimeter size).

In this review we focus on optical barcodes based on microcavities and microlasers. A microcavity confines the light to a small volume and produces optical resonances with well-defined frequencies and spatial intensity profiles. A microcavity can change the spectrum of the light which is reflected or transmitted through it, or it can modify the emission spectrum of a fluorescent material within. While any of the above can be used for barcoding, the case with the fluorescent material within the microcavity is the most frequently used. In this case, when gain material is contained inside, the microcavities can also be operated as lasers, i.e., above the lasing threshold. In this case the light is amplified by stimulated emission and if the gain is larger than the loss, then the microcavity operates in the lasing regime. The most typical characteristics of microcavities and microlasers are the narrow spectral lines in the spectrum. This enables the generation of a much larger number of barcodes in comparison to other spectral barcodes. Also, the narrow spectral lines can be more easily decoded in media with strong scattering, absorption and autofluorescence. The disadvantages of microcavity-based barcodes are their relatively large size (a micrometer or more) compared to, for example, a single fluorescent molecule (nanometers), and in the majority of cases the requirement for a high-resolution spectrometer for the readout. Recently, several reviews of microcavities and microlasers were published;^28−31^ however, they all focus more on sensing applications, while barcoding is not given much consideration.

This review is structured as follows. First, we consider graphical and spectral barcodes as the prevailing types of optical microbarcodes. Then, we present the types of microcavities and microlasers that have been used for barcoding (Fabry–Pérot microcavities, whispering-gallery-mode microcavities, random lasers, and distributed feedback microlasers) and discuss their general properties. After that, we describe the principles of barcoding with microcavities and microlasers. Different ways of encoding and reading out the barcodes are introduced, followed by a comparison of randomly generated barcodes and barcodes with predefined information encoding. We list different methods of multiplexing, used to increase the number of unique barcodes, and discuss practical considerations about the encoding and readout of the barcodes. We finish the section on barcoding principles with a short discussion of the multimodal use of microcavity barcodes. Then, we review experimental demonstrations of microlaser- and microcavity-based barcodes from the literature, listed by microcavity type. After that, we look at examples from the literature based on applications for which microcavity barcoding has been used, i.e., cell tagging and tracking, product labeling, and anticounterfeiting. We finish the review with a discussion of the future prospects for microcavity- and microlaser-based barcoding. There we discuss new types of microcavities and microlasers, as well as new ways of increasing the number of unique barcodes to further increase the amount of encoded information, and new applications the microcavity barcodes could be used for.

## Types of Optical Microbarcodes

We categorize the optical microbarcodes in three different encoding schemes: graphical, spectral, and others (Figure 1). Graphical and spectral barcodes are described briefly in this section. Other encoding techniques include polarization, lifetime,^20^ and angular momentum.^27^

### Graphical Barcodes

Similar to macroscopic classical barcodes and QR codes, graphical microbarcodes use a graphical pattern, such as linear sequences or 2D shapes to encode the information. Examples of graphical encoding include multifunctional encoded particles for high-throughput analysis,^32^ striped metal nanowire-based optical tags,^33^ multicolor barcodes in a single upconversion crystal,^34^ polymer-microparticle-based barcodes,^35^ and encoding microcarriers using spatially selective photobleaching.^36^ Their main advantage is the easy readout, since they can be read with common imaging equipment, such as a microscope and a camera. However, the barcode needs to be properly oriented in the focus of the optical setup and an unobstructed view is necessary for a successful readout. The size of such barcodes is also ultimately limited by the resolution of the optical setup. Submicrometer graphical barcodes have also been developed, but these require a super-resolution microscope for the readout.^37^

### Spectral Barcodes

The second type of optical barcodes is spectrum based, where all the information is encoded in the emission spectrum. In general, the readout does not require an imaging system, is rapid and does not depend on the barcode’s orientation. Fluorescence-based encoding is the most common technique, because of the easily tunable emission spectra, based on the structure of the fluorophore, and the easy readout. Examples include fluorescent-dye-doped micro- and nanoparticles,^38,39^ fluorescent nanorods,^40^ DNA-based fluorescence nanobarcodes^41^ and genetically encoded fluorescent barcodes for cellular multiplexing.^42^ Among the fluorescent materials, it is mostly organic dyes that are used for fluorescent barcodes, due to their widespread availability, but quantum dots and fluorescent proteins are also commonly used, especially in biological applications.^11,43^ The main disadvantages of fluorescent barcodes are the photobleaching and the relatively broad emission spectra, which limits their encoding capacity.

Instead of fluorescence, Raman scattering can also be employed as a signal for spectral barcodes.^21^ Raman barcodes have some prominent characteristics, such as good photostability, high capacity of the multiplexing and can be spectrally separated from fluorescence signals. The signal can, however, overlap with the background originating from other molecules in the sample.^21^ This can be solved by using novel Raman dyes that have a larger Raman shift, so that the signal does not overlap significantly with the background, and also provide a larger barcoding capacity.^44−46^ Spontaneous Raman scattering also has a relatively small signal compared to, for example, fluorescence. Surface-enhanced Raman scattering (SERS) is therefore used to increase the signal.^22,47−50^ Raman-based barcoding is less common than fluorescence-based barcoding.

A reflection spectrum can also be employed for barcoding. Most frequently, photonic crystals are used to create structural color encoding. The color can be tuned by changing the periodicity of the photonic crystals and their refractive index. Hence, photonic crystal barcodes with a unique structure can be used for sensing, anticounterfeiting, and multiplex bioassays.^51,52^ To generate a unique reflection spectrum, interference effects can also be employed, as in the case of reflections from a dielectric spherical particle.^53^

Microcavity- and microlaser-based barcodes are generally categorized as spectral barcodes, since the information is encoded in the spectrum. However, less often the information can also be encoded using other mechanisms, such as intensity, laser threshold, and polarization.

## General Properties of the Microcavity and Microlaser Types Used for Barcoding

**Figure 2:**
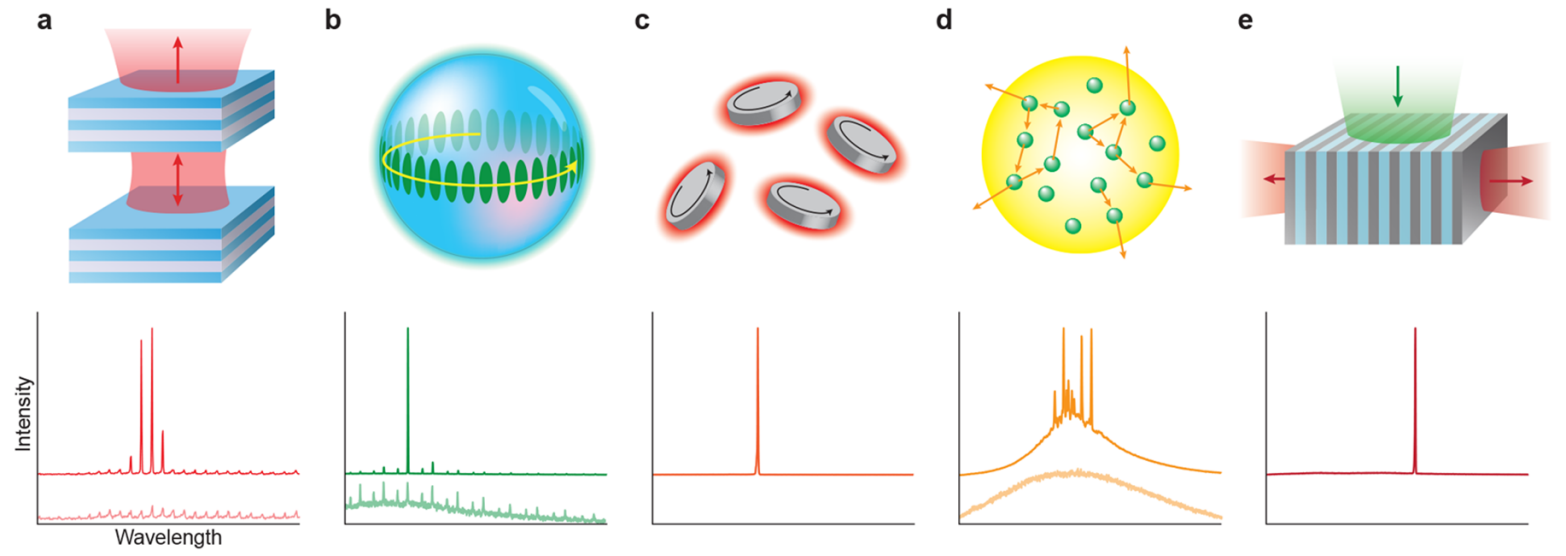
Schematics of different types of microcavities and lasers. For each type of microcavity, its lasing spectrum is shown in the bottom panel. In a, b, and d, the spectra below the lasing threshold are also shown in a lighter color. (a) Fabry–Pérot microcavity. Light bounces between two parallel flat mirrors. Peaks in the spectrum correspond to the wavelengths at which a standing wave forms in the cavity and constructive interference occurs. (b) Whispering-gallery-mode (WGM) microcavity. Due to the total internal reflection at the resonator’s boundary, the light circulates the cavity along its perimeter. Spectral peaks appear at resonant wavelengths, for which the optical path, traveled in a round trip, equals an integer multiple of the wavelength. (c) Microdisk—a special case of WGM microcavity. Due to its small size (submicrometer diameter), the consecutive resonant wavelengths are so far apart that only one falls into the spectral region of the optical gain. Therefore, microdisk WGM lasers exhibit single-mode lasing. (d) Random laser. The prolonged optical path is enabled by strong scattering in the gain medium. The spectral peaks appear due to the light propagating in close loops between the scatterers, resembling conventional optical cavities. (e) Distributed-feedback (DFB) laser. Light exhibits Bragg scattering on a periodic variation of the refractive index. The periodicity of the structure determines the emission wavelength.

Various types of microcavities and microlasers have been utilized for barcoding (Figure 2). In this section we describe their main characteristics that are related to barcoding. Most commonly, whispering-gallery-mode (WGM) microcavities and microlasers are used, either in the shape of microspheres or in the shape of microdisks, although Fabry–Pérot microcavities/lasers, random lasers and distributed feedback (DFB) lasers also have been used. In the future, other types of microcavity-based barcodes seem likely to appear for barcoding applications.

The design of the microcavity, in terms of its type and dimensions, is determined by the application. For some applications, a small size is of crucial importance, as the microcavity is to be fitted into a limited volume, while for other applications, it might be more important that the microcavity is hard to replicate.

The microcavity confines the light to a small volume, with typical dimensions in the range from below a micrometer to a few hundred micrometers.^54^ Its eigenmodes comprise a particular set of transverse and longitudinal cavity modes.^55^ The cavity shape and size in the lateral dimension determine the transverse modes, whereas the longitudinal modes are determined by the cavity dimension parallel to the direction of the light’s propagation. The number of cavity modes is proportional to the volume of the microcavity. For light of a certain wavelength to be resonant in a cavity, the optical path traveled in one round trip must be equal to an integer multiple *l* of the resonant wavelength. The resonant condition can be expressed as
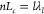
1where *n* is the effective refractive index inside the cavity, *L*_*c*_ is the round-trip cavity length, *l* is the longitudinal mode number, and λ_*l*_ is the resonant wavelength. The spacing between the wavelengths of two consecutive modes is called the free spectral range (FSR):
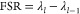
2

The fact that the resonances depend on the size of the microcavity and its refractive index makes it possible to create an abundance of distinct microcavities with distinguishable spectra, which can serve as barcodes.

The resonator’s performance is characterized by the quality factor *Q*, which is directly proportional to the lifetime of the cavity τ_*c*_, the time that the light stays trapped inside the cavity. Different cavity modes can experience different losses in the cavity, resulting in different *Q*-factors for each of the modes. In a passive cavity the *Q*-factor is inversely proportional to the resonance line width Δλ of a certain laser mode, as this is determined by the resonator’s losses:
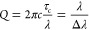
3Here, *c* is the speed of light and λ is the resonance wavelength.

If a cavity contains gain material, it can act as a laser. When pumped above the lasing threshold, population inversion is achieved in the gain medium, enabling the amplification of resonant light through the process of stimulated emission. Sharp laser lines emerge in the emission spectrum at the positions of the resonant wavelengths. Due to the cavity losses becoming compensated by gain, the line width decreases considerably. The overlap of the gain region with the resonant modes determines how many modes will be visible. If the gain region is narrower than the FSR, then only one mode will start lasing, resulting in a single-mode laser. Sharp spectral lines can also be present below the lasing threshold due to the Purcell effect in the case when the ratio between the *Q*-factor and the mode volume is large enough.

In microcavities used for barcoding, *Q*-factors can be very high. Typically, WGMs in microspheres and microdisks have Q factors in the range between 10^3^ and 10^6^.^56,57^ Thus, the production of a large number of different lasers that can be easily distinguished from each other is possible. This provides an efficient way of barcoding, in contrast to when fluorescence emission is used and only a few different colors can be reliably used as barcodes.

### Fabry–Pérot Microlasers

When laser cavities are concerned, we tend to think of a Fabry–Pérot (FP) cavity—a linear cavity, consisting of two mirrors with a gain medium between them,^55^ as drawn schematically in Figure 2a. Light bounces between the two mirrors, one of which is partially transmissive, and forms a standing wave. With every passing the light is amplified. In such a cavity, the lasing spectrum can be tuned by changing the gain medium, the cavity length, the mirror shape and by inserting objects into the cavity.

Apart from this classical representation a Fabry–Pérot-type cavity can also be realized as an (elongated) object made of a high-refractive-index material, with two parallel, flat surfaces acting as mirrors. An example of such a FP cavity is an organic single-crystalline waveguide, where the end crystal facets act as mirrors and the organic crystal as a whole acts as the gain material.^58^ Another example is a semiconductor nanowire, similarly forming a cavity and simultaneously acting as an active medium.^59−61^

### Whispering-Gallery-Mode Microcavities and Lasers

The whispering-gallery-mode microcavity is realized as a micro-object with a circular cross-section: a microsphere (Figure 2b), a microdisk (Figure 2c), a microring, etc.^55,62^ In order to confine the light, these micro-objects need a smooth surface and a larger refractive index than the material they are immersed in. In this way, due to total internal reflection at the surface of the microcavity, light is guided along the perimeter. The modes have either transverse electric (TE) or transverse magnetic (TM) polarization. They circulate close to the boundary and have an evanescent tail, extending out of the cavity. Through this tail the WGMs are coupled with the surrounding medium.^63^ In one round trip the light travels a distance of 2*πnR*, where *R* is the cavity radius and *n* its effective refractive index. As the WGMs circulate very close to the cavity boundary, the effective refractive index depends on both the internal and external refractive indices. WGM resonances are obtained at positions in the emission spectrum for which a resonant condition (2*πnR* = *lλ*_*l*_) is fulfilled. Small differences in the microcavity size, well under the diffraction limit, correspond to easily measurable spectral shifts. This is used in several applications of WGM microcavities—most notably in sensing. The positions of the spectral lines depend on three parameters: the microcavity size, the internal and the external refractive index. When one of these parameters is known in advance, the other two can be calculated by fitting the spectral position of the peaks to the equations describing the WGMs.^64−66^ Typically, the internal refractive index is fixed and known in advance. Fitting two parameters independently is possible because the TE and TM polarizations each have a different sensitivity to the external refractive index. In this case multimode emission is required. At least one TE and one TM mode and two consecutive either TE or TM modes must be visible. This is because the FSR between two consecutive TE or TM modes depends predominantly on the resonator’s size and the internal refractive index, whereas the difference between neighboring TE and TM modes depends predominantly on the ratio between the internal and external refractive indices.

When a gain material is present in the WGM microcavity or even only at its surface, lasing can be achieved. Below as well as above the threshold the WGM spectrum exhibits a series of narrow lines, corresponding to subsequent cavity modes. In microspheres the gain material is most commonly realized in the form of a fluorescent dye, semiconductor material or upconverting nanoparticles.^67^ Microspheres can in fact be dyed liquid droplets immersed in an immiscible medium, or they can be dye-doped solid spheres typically made from organic materials. The beads are nondeformable compared to the dyed droplets and are therefore more frequently used for barcoding.

Another type of WGM microcavity is microdisks, which can be made from a number of materials, but for barcoding they are most frequently made from semiconductor materials such as InAlGaAs, InGaAsP, and AlGaInP. The semiconductor material acts simultaneously as the microcavity and as the gain material. Due to the high refractive index of the semiconductors, high quality factors can be achieved and lasing can be observed in microcavities with submicron diameters. Because of their small size, the FSR is typically larger than the gain spectral region. Therefore, the microdisks support single-mode lasing.

### Random Lasers

Besides Fabry–Pérot and WGM microlasers, random lasers can also be used for barcoding. The prolonged optical path through the optical gain material in random lasers results not from reflections at the cavity edges, but rather from scattering, as shown schematically in Figure 2d. A random laser can be made in several different ways. For example, scattering particles can be inserted into a nonscattering gain material (e.g., silver particles dispersed in a dye solution^68^). In this case the lasing threshold depends strongly on the density of the scattering particles. Another way is to use a highly scattering material and label it with a fluorescent dye (e.g., fluorescently labeled scattering tissue^69^). A random laser can also be entirely made of a highly scattering material that acts as a gain material at the same time (e.g., semiconductor powder,^70^ neodymium-doped glass powder, ZnO nanorods or aggregates,^71^ semiconductor disordered photonic crystals^72^). While powders and nano/microparticles are obvious choices for the highly scattering materials, high scattering can also be achieved by carefully designing the gain material in disordered shapes, such as networks of nanoscale fibers made by electrospinning^73^ or different porous structures, such as photonic glasses and inverse glasses,^74^ and disordered photonic crystals.^72^

In random lasers the lasing can occur in two regimes—with incoherent or coherent feedback.^71^ In the first regime nonresonant feedback is provided by scatterers prolonging the optical path through the gain material. Above the threshold the emission bandwidth is narrowed as the most frequent photons with wavelengths close to the emission maximum are multiplied most rapidly through stimulated emission. The central wavelength of a random laser with incoherent feedback depends only on the gain spectrum; the width of the emission spectrum is decreased to a few nanometers.^75^ This regime is also known as the diffusive random-laser regime. It is present in systems with weaker scattering, where the optical modes are extended over the whole system. Different modes overlap spatially and spectrally, and are usually averaged either spatially or temporally. Incoherent random lasers can be used to generate a narrower emission compared to spontaneous emission and in this way they can increase the number of unique emission spectra and therefore the number of barcodes.

Coherent feedback in a random laser is realized as the photons in the scattering gain medium can return to the original position, thus traveling in a closed loop. This usually happens in a random laser with a strongly scattering medium, where the light is scattered frequently. The closed paths inside the scattering medium can be thought of as cavities that provide resonant feedback and contribute to strong interference, resulting in spatially localized modes (Anderson-localized random lasers).^75^ The spectrum of such a random laser contains sharp peaks. As every mode originates from a different part of the system—a different closed path between the scatterers—the direction of the laser emission depends on the exact position of the excitation. The sharp peaks that appear due to the coherent feedback might appear simultaneously with the overall spectral narrowing, as the two regimes of random lasing might both be present.^76^

Due to a rather complex output spectrum containing multiple spectral peaks, random lasers seem to be ideal candidates for barcoding. Additionally, in some cases the random lasers can be small, down to 1.7 μm.^77^ However, unfortunately the emission spectrum can change pulse to pulse as well as with the direction of observation. Therefore, not spectral peaks, but the threshold value has been employed for barcode encoding.^68,78^

### Distributed-Feedback Lasers

In distributed-feedback lasers the cavity is realized as a longitudinal periodic variation of the refractive index, either in the gain medium itself or in the material into which the gain medium is embedded (Figure 2e). On the periodic structure light exhibits Bragg reflection and is thus continuously being returned to the cavity. A DFB laser usually operates in single mode. The emission wavelength is determined by the periodicity of the structure and can be used as a barcode. A DFB laser can be made as a very thin membrane and attached to a variety of substrates.^79^

Another type of DFB laser is a chiral liquid-crystal laser.^80^ Here, rod-like chiral molecules twist helically along an axis, perpendicular to the molecular axis. Because of the birefringence of the liquid-crystal materials, the twist results in a periodic variation of the effective refractive index along the helical axis. In this type of laser the gain material is usually inserted into the liquid-crystal matrix as dye molecules.

## Principles of Barcoding with the Use of Microcavities and Microlasers

**Figure 3:**
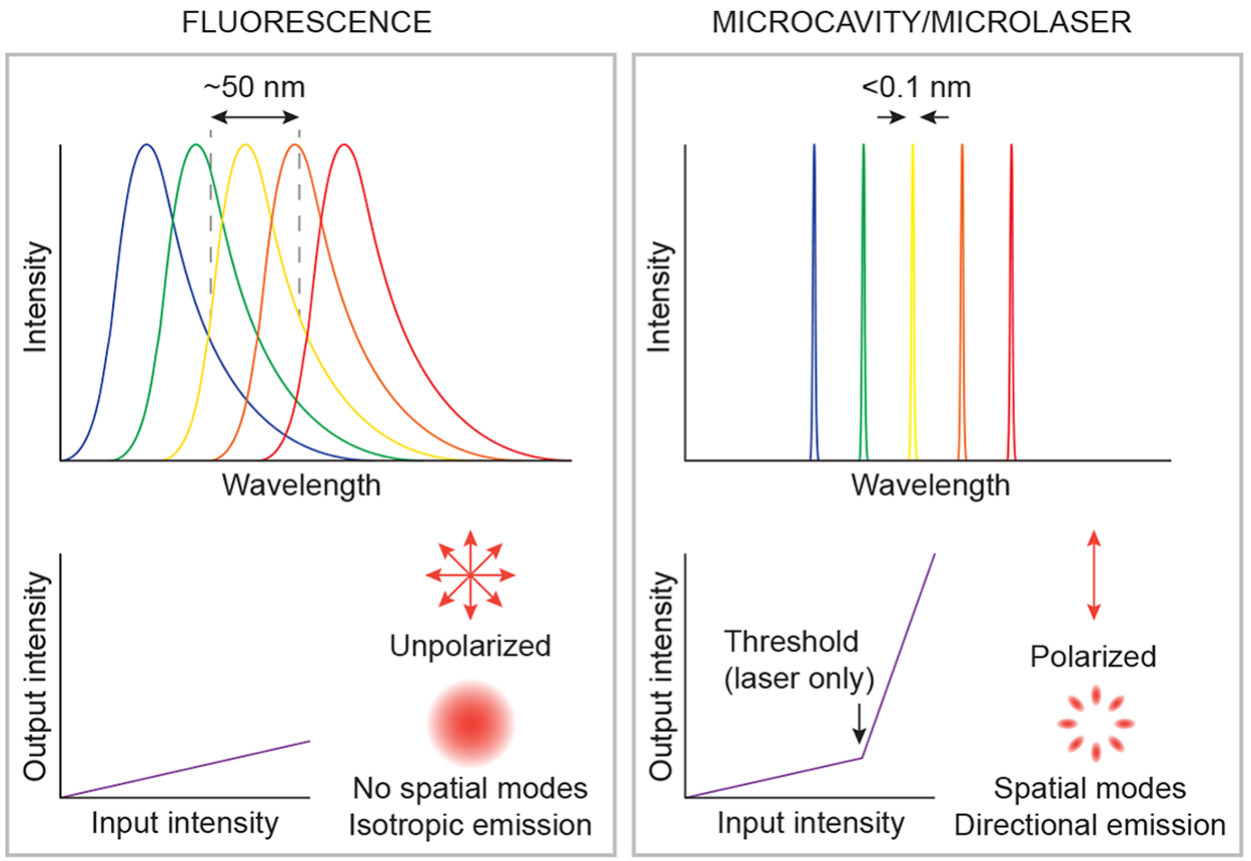
Comparison of the spontaneous fluorescence emission with the emission from a microcavity or a microlaser. The main differences are in the width of the emission spectrum, the nonlinearity of the output, the polarization, and the existence of spatial modes. The spectrum of spontaneous emission is at least 100 times wider than the emission of a microcavity, which results in the overlap of different sources, reducing the number of colors that can be used for the barcoding. Furthermore, lasers have a highly nonlinear output when the input power is increased; they typically have a polarized output and sometimes distinguishable spatial modes and a directional output.

For microcavity barcodes the information can be encoded in various ways in the properties of the emitted light, which is dependent on the properties of the microcavity, such as size, shape, and gain material. The goal is to generate as many unique barcodes as possible. Microcavities are ideal candidates for this since the cavity itself modifies the emission characteristics of the fluorescent gain material within (Figure 3). Most notably, while the fluorescence emission width is of the order of 50 nm, microcavities have a much narrower emission, frequently below 0.1 nm.^81^ Since the emission line width is several hundred times narrower, the number of distinguishable “colors” is larger by the same factor. Other emission properties also change when inserting a fluorescent material into the microcavity. In the case of lasers, the output intensity is highly nonlinear when increasing the input intensity and a clear lasing threshold is observed. Both below and above the lasing threshold, the emitted light can be polarized and well-defined spatial modes are present. The modes can be observed as a particular intensity pattern and as a directional output.

### Encoding and Readout of the Barcode

The output of a microcavity can be used in various ways to encode the actual barcode. The encoding variables can be, for example, the wavelength of a single or multiple spectral lines, the size of the microcavity (calculated from the spectrum), and the value of the laser threshold. Also, the barcode can be random, due to the random properties of the microcavities, or more rarely, it can be predefined, so that some useful information can be encoded.

**Figure 4:**
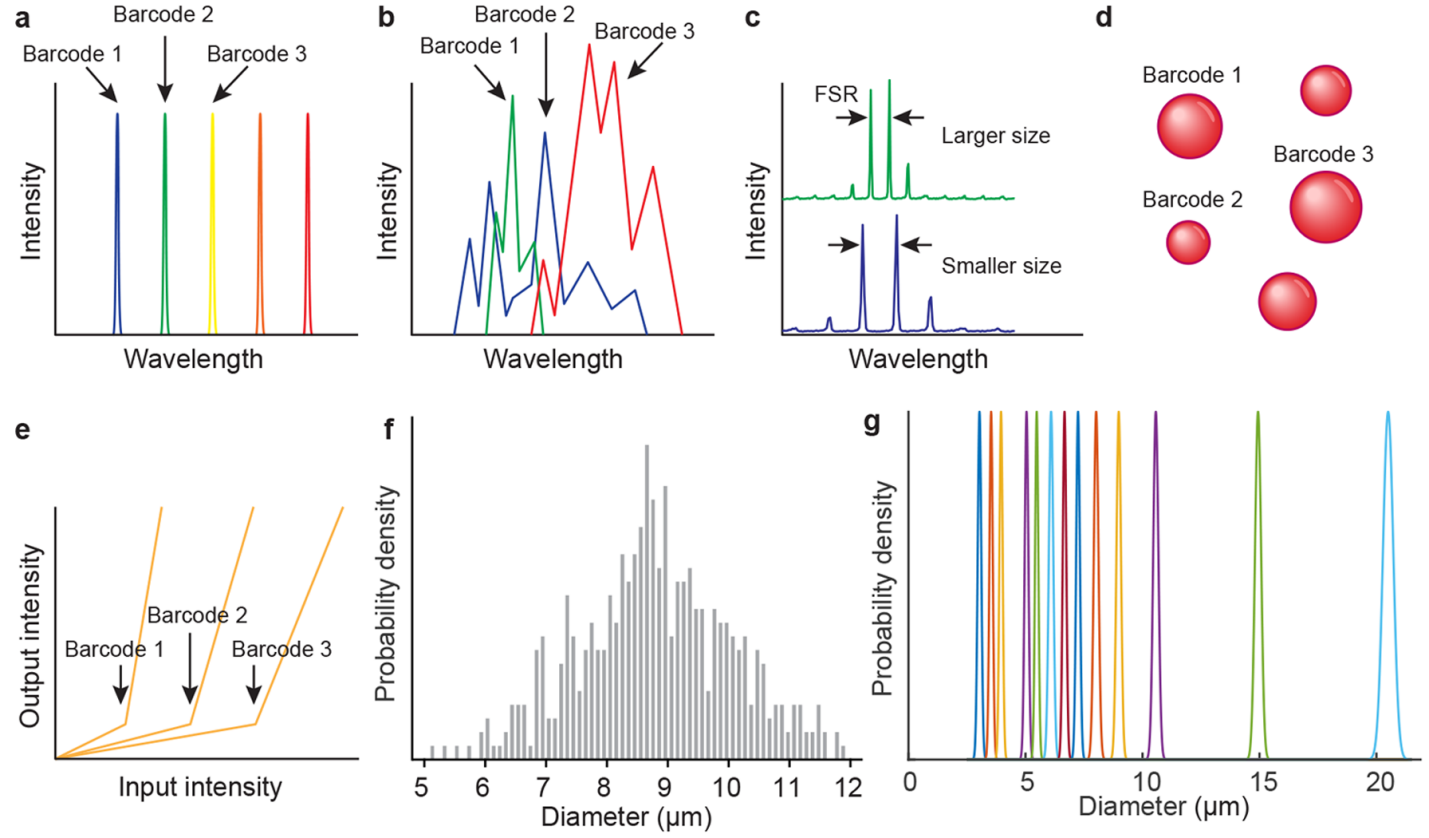
Principles of using microcavities for barcoding. (a) For single-mode emission, the wavelength of the spectral line defines each barcode. (b) For multimode emission multiple independent spectral lines can be used to define the barcode. (c) For well-defined cavities the modes have, for example, well-defined spacings, which are dependent on the cavity’s optical size. (d) The size or some similar property can be used as the barcode. (e) Laser threshold values can also be used to encode the barcode. (f) Size distribution of a commercial sample of polydispersed polystyrene microbeads, which can be used as random barcodes. (g) Size distributions of 13 samples of commercially available monodispersed microspheres, which can be used for barcodes with predefined information. An additional 7 sizes could be fitted in this range, resulting in a total of 20 distinguishable sizes.

The most obvious encoding choice is in the case of single-mode lasers, where simply the central emission wavelength is the barcode (Figure 4a). The most common single-mode microlasers are small WGM lasers where the FSR is large enough so that only one mode is within the gain region. The other common type of single-mode lasers is DFB lasers, where the emission wavelength is directly related to the periodicity of the structure. The maximum number of barcodes that can be generated is proportional to the wavelength range where the emission can be generated and inversely proportional to the width of the spectral peaks (either limited by the microlaser itself or by the detection system).

For multimode emission, the spectral lines can be used as unique identifiers in various ways (Figure 4b). Wavelength, width and the intensity of each spectral peak can be taken into account. For lasers with a complex cavity geometry, such as random lasers,^76^ the lasing peaks are relatively independent of each other, so the positions and possibly the intensities of all the peaks can be used as a barcode. We can imagine traditional barcodes, i.e., those that are composed of bars of different widths. Similarly, the position of the bars can be defined as the central wavelengths of the spectral peaks and the width of the bars can be defined as the fluorescence intensity of each peak. However, in most multimode cavities the modes are not independent of each other. For example, in a simple Fabry–Pérot the modes are equally spaced and the positions of all the peaks are defined by the optical length of the cavity (Figure 4c). Therefore, a single parameter, i.e., the size of the cavity, should be used as a barcode (Figure 4d). For more complex cavity geometries, more parameters influence the cavity modes and can be independently used as the barcoding quantities.

WGM microcavities are one example where size can be used as a barcode. The number of distinguishable unique sizes can be calculated for an example of commercially available polydispersed fluorescent polystyrene beads (Thermo Scientific, Fluoro-Max, mean diameter of 8.8 μm, coefficient of variation of the particle diameter, CV = 18%) (Figure 4e). We assume that two barcodes are different if the bead diameters are more than 1 nm apart, since typically the size can be calculated to better than 1 nm. The width of the particle size distribution in this particular case is calculated from the mean diameter and the CV is 3.2 μm. If the diameters are spaced perfectly in 1 nm intervals, this can give a theoretical maximum number of barcodes equal to 3200. In reality, since the sample contains beads with random sizes, two or more barcodes can be the same, which reduces the total number of unique barcodes. Also, the size distribution of any chemically produced spheres is not uniform (Figure 4e) and the chance of two barcodes being the same is larger at the center of the distribution. The actual number of unique barcodes for those containing either one or two beads with random sizes is given in the Multiplexing subsection.

Alternatively, instead of the spectrum or size, the pumping power at which the lasing threshold is reached can be used for barcoding (Figure 4f). The threshold is measured by increasing the pumping power until the microlaser starts to emit laser light. The threshold behavior is very typical of laser cavities and it is not exhibited by other optical probes. Therefore, it can be easy to distinguish it from the background. To measure the threshold, no spectrometer is needed, since only the intensity of the light is measured. Threshold barcoding is especially useful for counterfeiting, as described in the applications section.

### Random Barcode Generation versus Predefined Information Encoding

For the majority of microcavity-based barcodes that have been experimentally demonstrated so far, there is no predefined information encoded in the barcodes. Unique barcodes are generated through the inherent randomness of their manufacturing, such as the polydispersity in their size (Figure 4e). For most barcoding applications this randomness is desirable. On one hand, it facilitates the production of very complex barcodes that are practically impossible to replicate, which is favorable for anticounterfeiting applications. On the other hand, it enables the generation of a large number of unique barcodes. This is useful when many entities need to be tagged, for example, in cell tagging. However, if some information is to be encoded into the barcode, as is usually the case with macroscopic barcodes, then the manufacturing process needs to be accurately controlled. A limited number of cases have been demonstrated for the generation of predefined microcavity barcodes. The number of bits that can be encoded by one microcavity can be calculated using log_2_(*n*), where *n* is the number of unique barcodes that can be generated. A seemingly large number of unique combinations only gives a modest amount of encoded information. For example, 1000 unique spectra can encode only ∼10 bits of information.

There are several ways to make microcavities with predefined sizes. Relatively monodispersed, dye-doped microbeads, made out of various materials, are commercially available from a number of manufacturers. These can be directly used as WGM microcavities and lasers. Typical commercial microspheres used as a size standard have a CV of 1–3% for 10 μm diameter microspheres. In Figure 4g there are size distributions of 13 samples of polystyrene spheres, as provided by the commercial supplier. The range 3–20 μm is chosen here as an example because microcavities smaller than 3 μm have a low *Q*-factor and therefore very wide resonant peaks, while for microcavities above 20 μm the spectrum also contains the peaks of higher radial modes, which makes any measurement of the size from the spectrum more difficult. Although these microbeads are considered to be monodispersed, their size distribution is still large and only about 20 different sizes could be used in this size range without a significant overlap. With such a small number of unique possibilities, the encoded information is relatively limited. Alternatively, a microfluidics device can be used to produce microcavities and microlasers with a controllable size.^82,83^ Typically, the coefficient of variation for droplets produced with standard microfluidics setups is of the order of 1% and sometimes down to 0.1%.^84^ For example, in the size range 20–40 μm and with a CV of 1% it would be possible to generate ∼100 unique barcodes.

For the microcavities made using lithographic methods, such as the case of semiconductor microdisk lasers,^57,85^ they can be manufactured with different sizes. However, due to their small size, to encode some information, their manufacture would need to be extremely accurate. Therefore, predefined information encoding has not been demonstrated so far for microdisk lasers. A way of producing microcavities with extreme accuracy is to monitor the optical modes in real time, while at the same time changing the size of the microcavity to reach the required size. For example, semiconductor microdisk cavities have been tuned to picometer size using photoelectrochemical etching,^86^ and droplets have been made to nanometer precision by controlled oil injection.^87^ These methods are, however, relatively slow and therefore not scalable to large numbers of microcavities. For DFB lasers^79,88^ the periodicity can be changed easily and controllably, so these types of lasers are especially convenient for the manufacture of predefined barcodes. In the case when the laser threshold is used as a barcode, this threshold can be tuned via the gain and loss of the microcavity, so that various thresholds can be produced.^68,89^

For all the cases mentioned above, the number of predefined barcodes that can be controllably produced is relatively small. Therefore, to store any useful amount of information, multiplexing should be used, as discussed in the following subsection.

### Multiplexing

**Figure 5:**
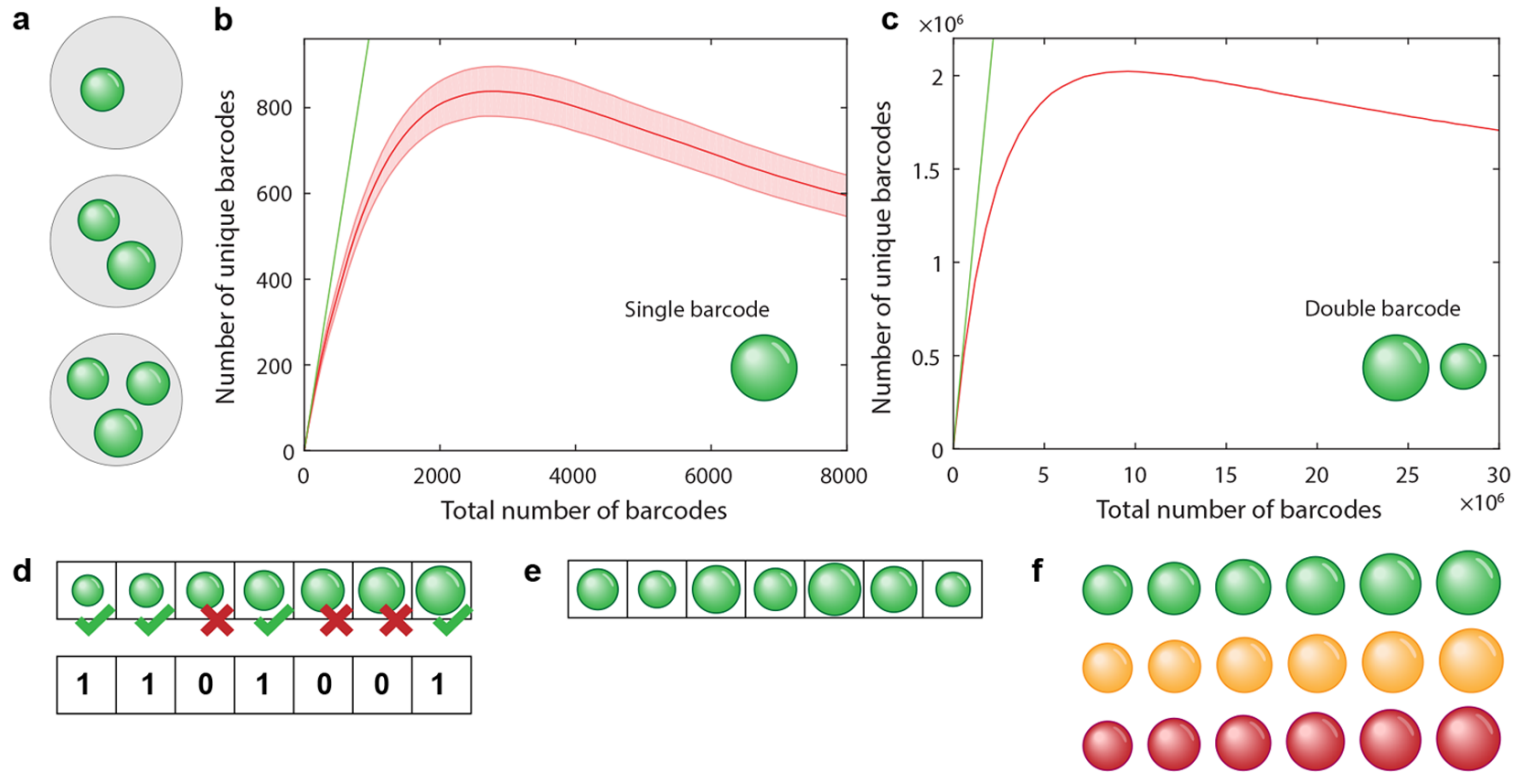
Principles of multiplexing. In all the panels the microcavity property that encodes the information is represented by the microsphere’s size, although other microcavity properties, such as the spectrum and threshold (and other microcavity types) can be used as well. (a) A single barcode can be composed of more than one microcavity, which greatly increases the number of possible unique barcodes. (b) Calculation of the number of unique barcodes when the total number of randomly picked barcodes is increased for barcodes containing one microcavity and (c) two microcavities. The green line represents the ideal case when all the barcodes are unique. The shaded area represents a 95% confidence interval. (d) Barcoding principle where any number of unique states can be selected to make a single barcode. This is in contrast to (a) where a fixed number of microcavities make up the barcode. In this case the barcode is encoded by the presence (“1”) or absence (“0”) of a certain state. (e) Microcavities positioned into a line—a simple example of combining microcavity-based and graphical encoding. (f) The number of unique barcodes can be increased by using two (or more) different properties of the microcavity, for example, different gain media (with different emission spectra) can be used in addition to using several microcavities of different sizes.

So far we have mostly described how a single property of a microcavity (e.g., the microcavity lasing wavelength and the threshold size) is used for the encoding. However, the number of unique barcodes can be increased via multiplexing, i.e., by combining two or more principles of encoding to generate a multidimensional barcode. Two or more microcavities can be joined together to generate a single barcode, or two different properties of a single microcavity (e.g., the emission wavelength and the lasing threshold) can be used to create a barcode. The microcavity-based encoding can also be combined with other optical barcoding techniques, such as graphical encoding. In this section we will discuss various ways of multiplexing, presented in Figure 5. Note that in the figure we show the microsphere size as the microcavity property used for encoding. The microsphere size is chosen for the purpose of clarity, as it is easily depicted. However, instead of the size of the microspheres, different microcavity properties (as well as different types of microcavities) can be used in actual cases.

The most obvious way to increase the number of unique barcodes is to use several microcavities together to create one barcode (Figure 5a). As the order of microcavities is usually not important, the number of unique combinations (*N*) is calculated as
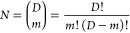
4where *m* is the number of microcavities, and each microcavity can exist in one out of *D* possible unique states (e.g., number of unique emission spectra or number of unique sizes). Here we assume that the barcode containing several microcavities is read by taking a single spectrum. From it, it is impossible to deduce how many microcavities with the same spectrum are present in the barcode. Therefore, the equation for the number of combinations without repetition is used. For example, if five microcavities are used and each microcavity can be in one out of 100 possible unique states, then the number of combinations is 7 × 10^7^. The number of unique barcodes increases rapidly with a larger number of microcavities in the group. Consequently, this strategy could be employed for multiplexing applications that require a huge number of unique barcodes.

However, since in most cases the sizes of the microcavities are random, there is a probability that two barcodes will be the same. As the total number of microcavities is increased, this probability also increases. If the barcode contains multiple microcavities, then the probability that such a barcode is identical to some other barcode is much lower than for barcodes containing only one microcavity. To quantify this, the number of unique barcodes for barcodes containing either one or two microcavities was calculated as a function of the total number of barcodes (Figure 5b,c). A random sample of beads with a Gaussian size distribution was generated (mean diameter = 8.8 μm, CV = 18%) and the number of unique barcodes was counted (for barcodes containing one microcavity, see Figure 5b). When the number of barcodes is small, the probability of two barcodes being the same is small, so the number of unique barcodes is approximately equal to the total number of barcodes (green line). When the number of barcodes increases, more and more of them will be of the same size, so the number of unique barcodes will decrease. In the limit of a very large number of barcodes, no barcode will be unique any more. The maximum number of unique barcodes of 830 is reached at 2800 total barcodes in the sample. Because of the random size distribution, the number of unique barcodes can fluctuate in each experiment. For example, even with a small number of beads there is still a chance that all the beads will have the same diameter. These fluctuations in the number of unique combinations are drawn as a 95% confidence interval. The number of unique combinations was also calculated for barcodes containing two beads (Figure 5c). The trend is similar to the single-bead case, although the number of unique combinations is much larger. The maximum number of unique barcodes with two beads is 2.0 × 10^6^ and is reached for 9.6 × 10^6^ total barcodes in the sample. Because of the large number of barcodes, the confidence interval is much smaller.

In the above-mentioned method of creating a barcode from several grouped microcavities, the barcode consists of a fixed number of microcavities (*m*), which can each occupy one of all the possible states. In this case two distinguishable barcodes contain the same number of microcavities, but these are in different states. There is another frequently used way of creating barcodes from more than one microcavity. Here, the number of microcavities is not fixed. Information is encoded through the presence (“1”) or absence (“0”) of certain values of a chosen microcavity property (e.g., a certain size, a certain lasing emission wavelength, a certain lasing threshold) in the sample, as shown schematically in Figure 5d. The number of unique barcodes *K* can be calculated as
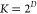
5where each microcavity can exist in one out of *D* possible unique states. For example, in the case of commercially available monodispersed microspheres, presented in Figure 4g, where 20 different sizes of microspheres could, in principle, be distinguished, approximately 10^6^ different barcodes can be encoded in this manner.

Another commonly used method for multiplexing is a combination of microcavity-based encoding and graphical encoding. Namely, the microcavities can be arranged in a particular graphical pattern. Already having multiple microcavities in an ordered group (e.g., ordered in a line, Figure 5e) serves to increase the number of unique barcodes. The number of unique barcodes (*M*) is calculated using the equation for the number of permutations without repetition as
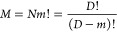
6The number of unique barcodes is larger by a factor of *m*! compared to the case when the order is not important (eq 4). Here, we again assumed that no repetitions are allowed. However, since the optical system allows us to read the order of the microcavities, they can also be repeated. In this case the number of unique barcodes is
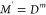
7As an example, let us take five microcavities, each being in one out of 100 possible unique states. In the case they are ordered but no repetitions are allowed, there are 9 × 10^9^ unique barcodes, whereas if repetitions are allowed, the number of unique barcodes is slightly higher at 10^10^.

Instead of being ordered into a line, microcavities can also be positioned into a two-dimensional pattern, creating a graphic pattern. In terms of the number of unique barcodes, this case is equivalent to the microcavities being ordered in a line. However, when a large number of microcavities is used to produce a barcode, for some applications it might be beneficial to use a more compact 2D shape rather than a long line.

Multiplexing can also be achieved by combining two or more properties of the microcavities. For example, different gain media (with different emission spectra) can be used in addition to the microcavity sizes, to increase the number of unique barcodes (Figure 5f). Multiple gain materials can be used simultaneously, and these can be independent of each other or they can interact, for example, via Förster resonance energy transfer (FRET).^90^ Other combinations of microcavity properties such as spectrum, size, peak positions, peak intensities and lasing thresholds can also be used simultaneously.

### Practical Considerations about Encoding and Readout of the Barcodes

To exploit the microcavity barcodes to their full potential, care must be taken in all the steps, including barcode design, excitation, readout and signal analysis. In this section practical considerations regarding all these steps are described in detail.

Most microcavity barcodes are spectrum based, so a spectrometer should be used. In most cases of spectral barcodes, no imaging system is needed, and even placing an optical fiber close to the microcavities is enough to excite them and collect enough light to send to the spectrometer. Imaging is, however, required in the case of multiplexing, where the order of the barcodes is also important, for example, by encoding with graphical patterns. The spectrometer’s resolution determines how close two spectral lines can be and remain resolvable. The microcavities used in barcoding are typically small, so the FSR is large and we do not need to resolve closely separated spectral lines. For the barcoding only the central position of the spectral line is important. The accuracy of the central position’s determination can be much better than the spectrometer’s resolution. This is, for example, achieved by fitting the spectral peak to a Lorentzian function or by calculating the center of mass to determine its position very accurately. As an example, using a spectrometer with a resolution of 0.05 nm it is possible to measure the central wavelength of a high signal-to-noise ratio WGM laser line to an accuracy of 0.001 nm.

A different situation occurs when there are multiple microcavities being read at the same time. For example, in the case of multiplexing. In such a case the spectral peaks can be close to each other and a higher spectral resolution is needed to resolve the peaks. The minimum distance between two peaks, so that they can be distinguished, is dependent on the peak’s width and the spectrometer’s resolution, whichever is smaller. In the case of the simultaneous readout of two or more multimode microcavities, multiple peaks can overlap, which makes the analysis more complicated. For example, it has been shown that up to five different WGM microcavities can be identified from a single spectra^65^ by using a spectral unmixing algorithm. From a single spectra the sizes and external refractive indices of five microcavities were accurately determined. The unmixing algorithm is based on the fact that the WGMs are equally spaced in terms of frequency. It searches for equally spaced peaks and groups them by FSR, which results in the separation of peaks originating from different microcavities.

To excite the laser-based barcodes above their lasing threshold, a pulsed nanosecond laser is normally used. While these lasers can be easily employed for research, industrial or medical applications, they are still too expensive for consumer use. For barcodes operating below the laser threshold, cheaper continuous wave lasers or LEDs can be used. A filter should still be used to filter out the excitation light and only let through fluorescent light.

An important consideration is the temporal stability of the barcode due to external factors, such as the change in the external refractive index, temperature, mechanical force, etc. All the microcavities where part of the optical field reaches out of the cavity into the surrounding medium are sensitive to changes in the external refractive index. This is, for example, the case in WGM microcavities and nanowire lasers. While this is good for sensing applications, it might change the barcode. One solution is to separate two unique barcodes in such a way as to accommodate the expected changes in the refractive index. As an example, the sensitivity of semiconductor microdisk lasers is 80 nm/RIU.^85^ For a refractive-index change of only 0.1 RIU the wavelength shift is 8 nm, which is almost 10 times more than usual spacing of two barcodes (1 nm). Therefore, this reduces the possible number of barcodes by a factor of 10. Another solution is to coat the microcavities with a few-100 nm-thick layer of transparent material to shield it. Yet another solution is to actually measure and then subtract the effect of the refractive-index change. This is possible if two optical modes of the same microcavity have a different sensitivity to the external refractive index. A typical example is WGM microcavities where it is possible to calculate both the microcavity size and the ratio between the internal and external refractive indices by taking into account the two polarizations (TE and TM). Therefore, the calculated size of the microcavity is independent of the external refractive index and is used as the barcode.

The temperature can change both the size and refractive index of the microcavity, and therefore the output. Based on the temperature variation that is to be expected for a particular application, the spacing of two unique barcodes has to be large enough to accommodate these changes. Furthermore, a material or combination of materials can be chosen for the microcavity to minimize the effect of the temperature. For example, for polystyrene the coefficient of the linear expansion is almost opposite to the temperature coefficient of the refractive index. Therefore, these two effects almost cancel each other out, resulting in a very small spectral shift of 3 pm/°C for a 10 μm microcavity immersed in water.^91^

The barcodes that contain organic dyes can exhibit photobleaching upon repeated reading or just from the ambient light. The wavelengths of the spectral peaks, however, do not change upon photobleaching, so this does not change the barcode; it just decreases the available signal. However, if the barcode is also encoded in the intensity or laser threshold, the photobleaching could be a problem and has to be taken into account. The threshold is particularly sensitive to the gain and loss of the microcavity. Therefore, it is also sometimes used for sensing purposes.^81^ The gain and loss can be changed not only by photobleaching, but also by the geometry of illumination, so in this case particular care has to be taken in the design of the illumination.

The directionality of the barcode output should also be considered. This is because laser output can be very directional. Depending on a particular application of the barcode it can be desirable for the light to be emitted in a particular direction, for example, to be able to read the barcode at a larger distance. In other instances the specific directionality of the laser emission can reduce the reliability, especially when the orientation of the laser particles changes with respect to the optical reader. For spherically symmetric microlasers, such as WGMs and onion Bragg lasers, the emission is isotropic. However, many other microlasers, such as DFB lasers, have at least partially directional emission. If omnidirectional emission is desired, light scatterers can be introduced into the laser or onto the surface. For example, microdisk lasers exhibit in-plane emission.^92^ To make microdisk lasers with omnidirectional emission, defects that scatter laser light in all directions were introduced, either at the cavity’s perimeter (increased surface roughness and/or dents or bumps) or at the cavity’s top and base (nanoparticle coating).^93^ For lasers of a size close to or less than the wavelength of light, the diffraction of light alone due to their small size makes the emission more omnidirectional. For example, nanowire lasers, even though they are highly asymmetrical, they are usually very thin, which results in a relatively isotropic emission.^94^

### Multimodal Use of Microcavity Barcodes

Microcavity barcodes can be simultaneously employed for some other application, such as sensing or imaging. It is, however, important that the barcode does not change over time, even if the same microcavity is used, for example, for sensing. For instance, in a typical sensing setup where a single molecular layer (or less) is bound to the surface of a WGM microcavity, the spectral shift is usually well below 1 nm,^28^ which would typically not affect the barcode. For multimode WGM microcavities the size and the external refractive index can be simultaneously extracted from their spectrum, as described earlier. Therefore, size can be used as a barcode, while the external refractive index can be used for sensing. All the microcavity barcodes also scatter light, so they can be used in any imaging modality that uses scattered light, such as bright-field imaging and optical coherence tomography.^60,61^

Apart from using the optical properties, the physical particle itself can be used simultaneously as a carrier for different active molecules. For example, the microcavities can be coated with fluorescent probes, which are sensitive to a particular substance or external factor. The change in intensity can, for example, be due to the binding of fluorescent molecules to the surface of the microcavity.^90^ If the emission spectrum of the microcavity and the fluorescent probes do not overlap, both the sensing and the barcoding can be carried out independently using the same particle.

## Experimental Demonstrations of Microlaser- and Microcavity-Based Barcodes

### Barcoding Based on Whispering-Gallery-Mode Microcavities and Lasers

WGM microcavities are the most-often used form of microcavities for barcoding. They are typically of a small size, have a high *Q*-factor and are easy to fabricate. They can exist in various forms, including organic and inorganic microspheres, microdroplets, and microdisks.

Several authors report on using commercially available polydispersed dye-doped polystyrene microspheres with diameters ranging from 10 to 20 μm.^56,65,91,95,96^ The microbeads contain fluorescent dye as the optical gain medium. Such microcavities can be used either below or above their lasing threshold. Usually, the microcavity size, calculated from the spectrum, is used as a barcode. For polystyrene microspheres containing green dye, the minimum size required for lasing is ∼10 μm. The microcavity’s diameter can be calculated to an accuracy of a few nanometers. Therefore, for a diameter range of 10–20 μm and a step size of 5 nm, approximately 2000 unique barcodes can be generated.

Alternatively, the spectrum itself or other WGM characteristics can be used as a barcode, for example, the dominant lasing peak wavelength (λ_max_) and the wavelength spacing between two TE peaks (Δλ).^95^ In this case the emission wavelength λ_max_ of all the microspheres was in an 8 nm-wide spectral range and Δλ was varied by approximately 2 nm. The small uncertainties in measuring λ_max_ and Δλ were 0.3 and 0.03 nm, respectively. For two barcodes to be distinguishable, they have to differ spectrally by more than twice the measuring uncertainty. Therefore, it would be possible to generate nearly 1800 unique tags in the described manner.

In general, there is a huge variety of commercially available microspheres made from different materials with the addition of different dyes. These different dyes can be used for multiplexing to increase the number of unique barcodes. Therefore, for someone wanting to make a simple barcoding experiment, buying microspheres is the best starting point.

**Figure 6:**
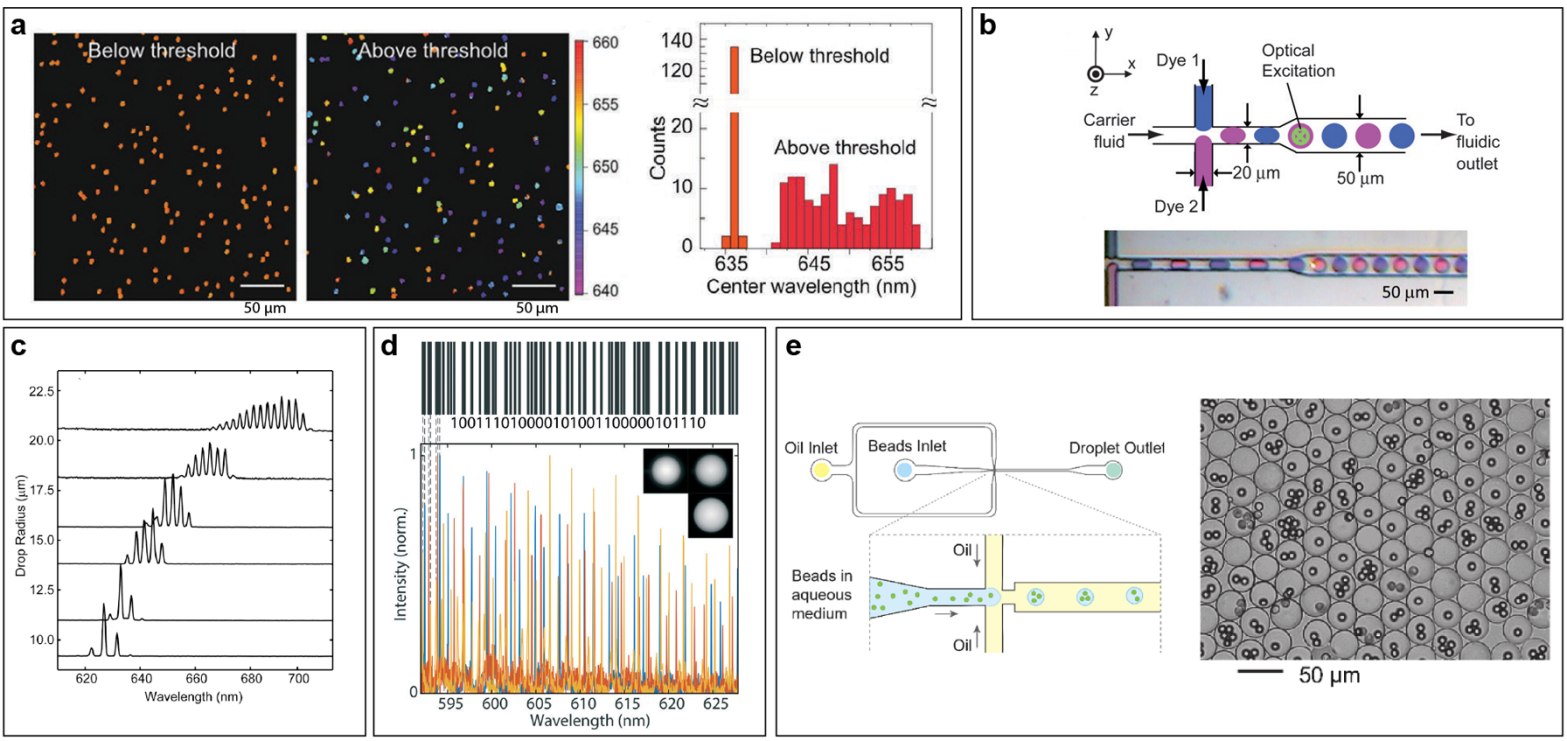
Experimental realizations of spherical WGM-based barcoding. (a) Quantum-dot microbeads above and below the lasing threshold, with color representing the central wavelength of the emission. The right-most panel shows the distribution of central wavelengths for 140 quantum-dot microbeads both above and below the lasing threshold. (b) Schematic of the droplet-generator setup having two opposing T-junctions for two different dye solutions, and a snapshot of the generation of two different types of droplets containing either Rhodamine 560 (pink) or Oxazine 720 (purple). (c) Lasing spectra corresponding to droplets with different radii, ranging from 21 to 7 μm (baseline of each spectrum represents the radius of the droplet). (d) Demonstration of the encoding of the word “STEFAN”, written as a binary number, using three microbeads with predefined sizes. (e) Generation of barcodes containing multiple microcavities by encapsulating fluorescently doped microspheres into larger hydrogel beads. Image credits: (a) Reprinted with permission from ref (97). Copyright (2021) Wiley-VCH GmbH. (b) Reproduced with permission from ref (83). Copyright The Royal Society of Chemistry 2009. (c) Reprinted with permission from ref (82). Copyright (2011) The Optical Society of America. (d) Reproduced with permission from ref (87). Copyright (2020) The Royal Society of Chemistry. (e) Reproduced with permission from ref (98). Copyright (2021) Optical Society of America.

When a WGM microcavity’s size is sufficiently reduced, the microlaser can exhibit single-mode emission. A microbead, made of quantum dots, bound together by ligands, is an example of such a single-mode microlaser.^97^ Compared to organic microspheres doped with organic dyes, such lasers have an increased refractive index, optical gain, and stability. In this example, single-mode operation is enabled by a high refractive index of the quantum dots and the small size of the cavity (down to 1.5 μm). The multicolor images of these microbeads above and below the lasing threshold are shown in Figure 6a. Below the lasing threshold, the microbeads exhibit photoluminescence with a central wavelength at approximately 636 nm, with a variation of only 0.2 nm. Above the lasing threshold the lasing peaks appear in a wider wavelength range of 640–658 nm, since the lasing wavelength depends on the size of the beads, which varies from bead to bead. The lasing line width is 0.6 nm, which enables 19 different wavelengths to be distinguished with a step of 1 nm. In the future, by multiplexing using quantum dots with several different emission spectra (in the range of 400–1600 nm) more than 1000 unique barcodes could, in principle, be possible.

In addition to solid microbeads, the spherical microcavities are frequently realized as droplets, dispersed in a liquid medium. Figure 6b shows an example of droplet-microlaser production by microfluidics, making possible the generation of droplets with varying diameters (20–40 μm) and alternating gain media (two dyes with different emission spectra), at a frequency of 3.6 kHz.^83^ In another example, 21 μm droplets were generated with a microfluidic device, with their size decreasing as they travel through the microfluidic channel, slowly dissolving in the outside medium.^82^ The changing droplet size resulted in a considerable wavelength tunability in the 620–700 nm range (Figure 6c). Both the free spectral range and the average lasing wavelength changed depending on the size of the droplets. The free spectral range is inversely proportional to the microcavity size as expected. The average lasing wavelength is dependent on both the gain and the loss. The gain is dependent only on the dye and not on the size of the microcavity, while the loss is also dependent on the radiative loss, which decreases with the increasing size of the microcavity. For very large microcavities the radiative loss is negligible, so that it is mostly the properties of the gain material that influence the average lasing wavelength. However, for smaller microcavities the radiative loss is more important, and since it is smaller for shorter wavelengths, the lasing is blue-shifted. From the barcoding perspective, both the free spectral range and the average lasing position are dependent on the size of the microcavity. Therefore, they cannot be used as two independent barcoding quantities.

Although in both of the above cases the droplets were not generated for the purpose of barcoding, they still demonstrate the possibility of creating droplets with controllable WGM lasing emission, by variable sizes and multiple dyes. The droplets can also be made out of a polymerizable material, so that solid beads can be created.^99^

The generation of monodispersed microdroplets and microbeads has also been demonstrated by real-time control and monitoring of the droplet size via WGMs, reaching nanometer accuracy.^87^ The achieved accuracy is two-to-3 orders of magnitude better than with the microfluidic techniques. Microdroplets were generated in a controlled manner at the tip of a microcapillary immersed in an external fluid by varying the pressure in the microcapillary. Size of the microsphere produced was in the range 20–40 μm, in which it is possible to generate 20,000 unique sizes with an accuracy of 1 nm. By utilizing three such microspheres together, 1.3 × 10^12^ combinations can be generated, which is equivalent to 40 bits of information, making it possible to encode numbers, such as the time and the date as well as short words. For example, the word “STEFAN” was encoded by using three beads with predefined sizes (Figure 6d).

To generate a barcode containing several microcavities, these need to be physically bound together. Such binding can, for example, be achieved by encapsulating multiple fluorescently doped microsphere cavities of random diameters into larger hydrogel microbeads, produced by microfluidics (Figure 6e).^98^ In this case, the hydrogel microbeads contain a random number of microcavities. The average number of contained microspheres can be controlled or tuned by varying the size of the hydrogel particles (by varying the microfluidic flow) and by varying the concentration of the microcavities in the aqueous mixture used for bead production. At a droplet-generation rate of 2.5 kHz, an estimated 10^5^ unique barcodes can be produced in less than 3 min.

**Figure 7:**
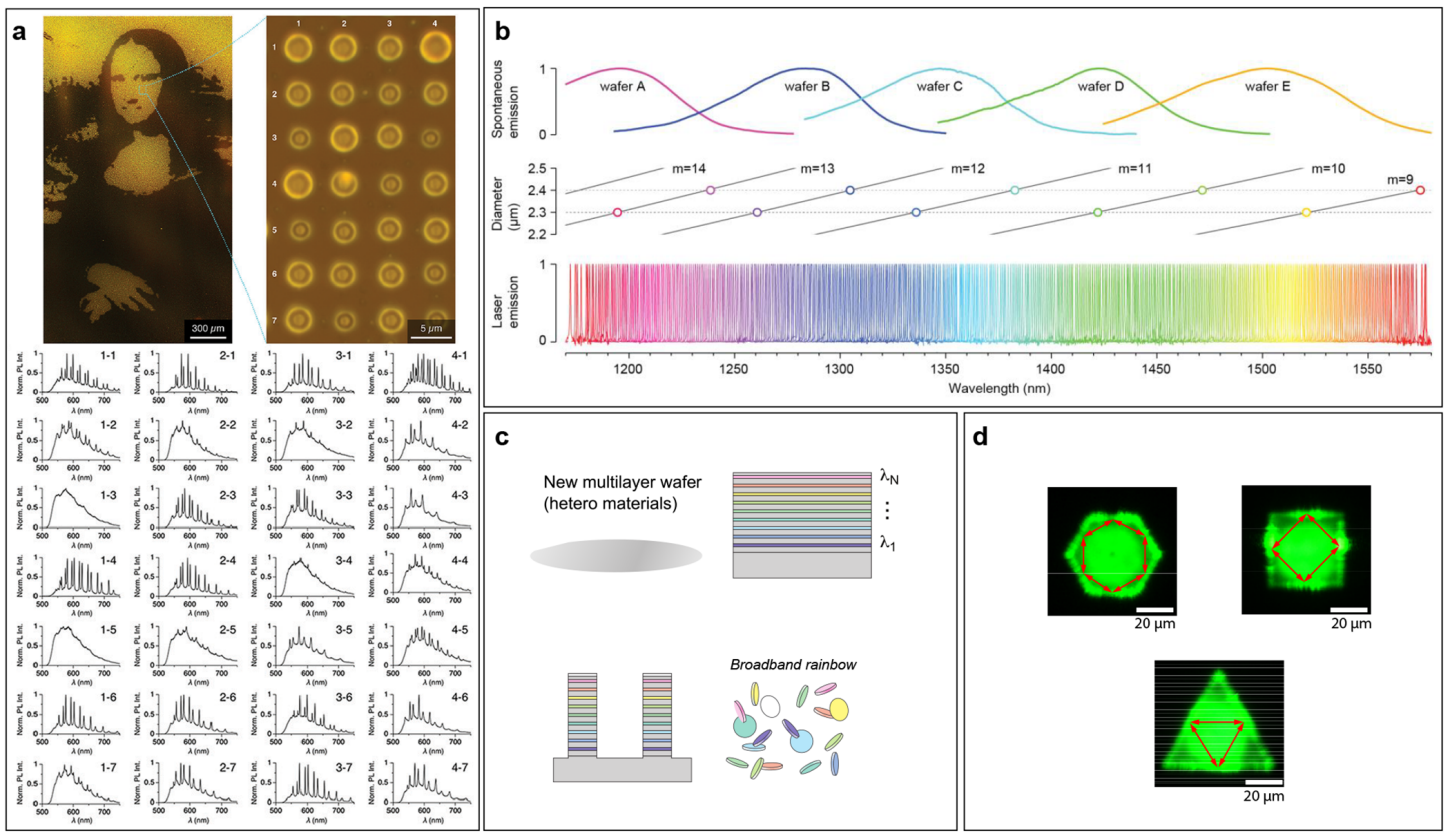
Experimental realizations of disk-shaped WGM-based barcoding. (a) Fluorescence micrograph of a micropainting that consists of an array of microhemispheres. Their corresponding emission spectra are shown on the bottom. (b) Laser emission from microdisk lasers (bottom) in relation to an optical gain corresponding to five different semiconductor wafers (top) and the size of the microdisks (middle). (c) Production of multicolor microdisk lasers through a multilayer epitaxial process enables the single-step generation of a large number of different barcodes. (d) Perovskite microdisks in three different shapes: hexagonal, tetragonal, and triangular. Each shape has a slightly different emission and laser threshold. Image credits: (a) Reproduced with permission from ref (100). Copyright (2020) The Royal Society of Chemistry. (b) Reproduced with permission from ref (85). Copyright (2019) Springer Nature. (c) Reproduced with permission from ref (101). Copyright (2021) American Chemical Society. (d) Reproduced with permission from ref (102). Copyright (2021) American Chemical Society.

Microcavities can also be arranged in complex 2D patterns, as shown in Figure 7a. In this case, the microhemispherical cavities were assembled via surface self-assembly on a micropatterned substrate, resulting in an array of millions of pixels/cm^2^, each exhibiting a different WGM spectrum.^100^ In this way a virtually unlimited amount of information can be encoded, although at the expense of needing an imaging system to measure the spectra from each pixel.

Another type of WGM microcavity frequently used in barcoding is semiconductor microdisk lasers. Due to their small size, they exhibit single-mode emission. They are made from III–V semiconductor materials, e.g., InAlGaAs or InGaAsP, for emission in the infrared^85^ and GaInP/AlGaInP for emission in the visible part of the spectrum.^57^ The infrared microdisk lasers measured approximately 2 μm in diameter and had a thickness of 400 nm. Such a small size is possible due to the fact that the semiconductor materials have a high gain and a high refractive index. The microdisks were also coated with silica using a solution-based process to make them biocompatible. When pumped with a 1064 nm laser, they exhibited lasing emission with a line width of 0.4 nm. Due to a slight variation in the sizes of microdisks, the lasing wavelength of the microdisks from a single wafer spanned across a 100 nm range—the change in microdisk diameter of 2 nm resulted in approximately 1 nm variation in the lasing wavelength. To extend the emission wavelength’s range, more semiconductor wafers with different chemical compositions (and therefore different fluorescence emission spectra) were used for the microdisk production, as shown in Figure 7b.^85^ For a given diameter, only 1 or 2 modes lie in the range of the gain bandwidth of each semiconductor wafer. Consequently, microdisks with a single-mode lasing emission in an ultrawide spectral range of 1170–1580 nm can be produced, as shown in Figure 7b, resulting in 400 unique barcodes. In these cases the variation in the microcavity size is random; however, it has also been shown that a continuous variation in sizes can be produced on a wafer,^57^ but the encoding of information has not been demonstrated so far.

Similarly, a novel method for the efficient production of microdisks with a variable laser wavelength spanning the range 1150–1650 nm was recently introduced.^101^ By using compositionally graded quaternary material to form a multilayer epitaxial structure, a rainbow of laser particles with emission wavelengths approximately covering a 500 nm range could be produced from a single parent wafer, as shown in Figure 7c.

Single-mode nanodisk lasers were also manufactured from epitaxially grown AlGaInP quantum-well structure.^57^ The nanodisk lasers were submicrometer in size, down to 700 nm in diameter and a thickness of 260 nm. They were pumped at 473 nm with sub pico-joule pluses and emitted light in the visible part of the spectrum at 660 nm. Their lasing emission could be tuned in a 40 nm range by varying their size.

Apart from microspheres and microdisks, WGM microcavities can also take more exotic shapes. One example of this is geometry-programmable perovskite microlasers.^102^ In this case, perovskite microdisks in three different shapes (hexagonal, tetragonal and triangular) were manufactured using the lithographic template-confined crystallization method (Figure 7d). Depending on the shape, the microlasers had a different laser threshold, which was the property employed for encoding the barcode. In the future, more shapes could be used to create even more distinct laser thresholds.

### Barcoding Based on Fabry–Pérot Microlasers

**Figure 8:**
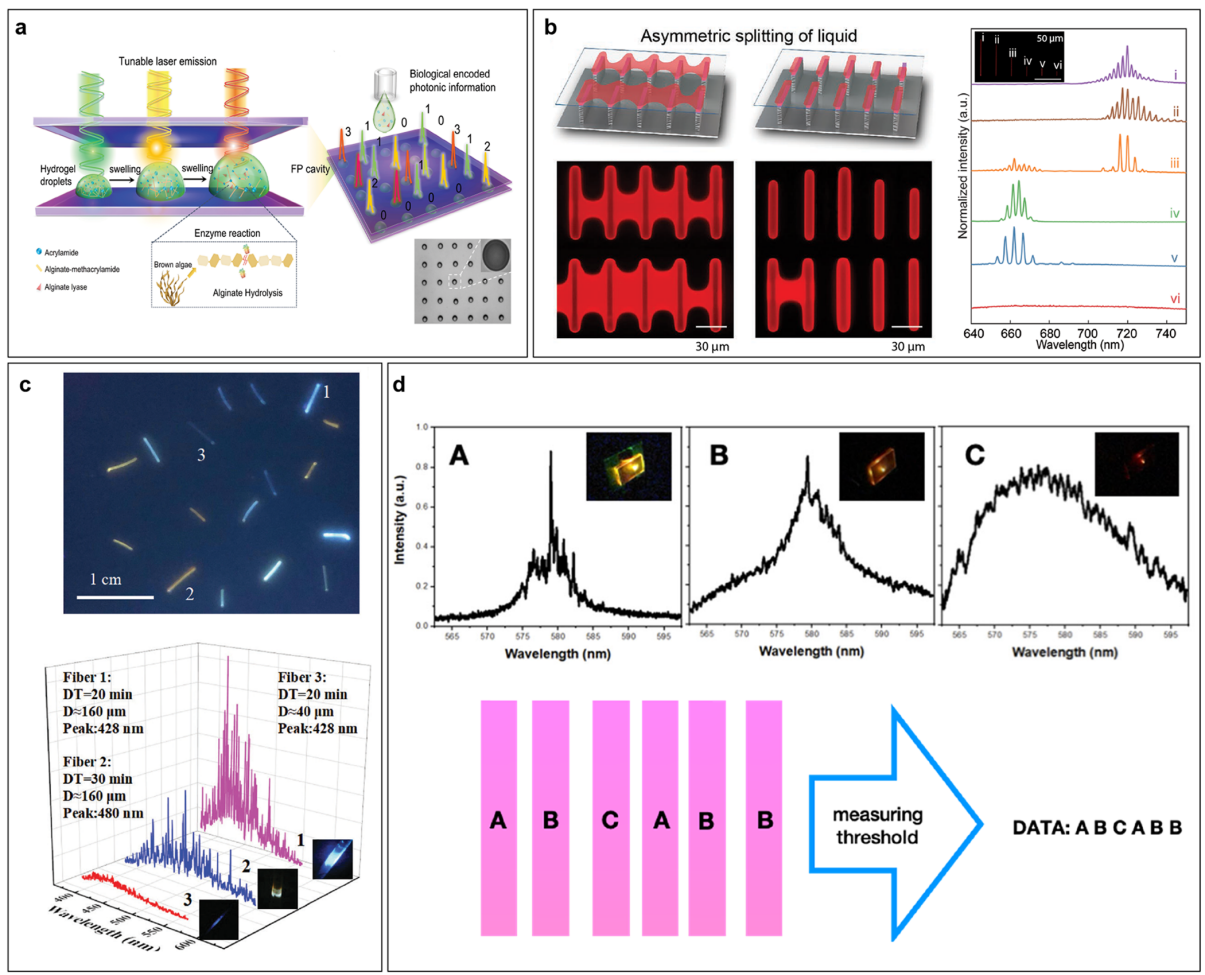
Experimental realizations of Fabry–Pérot microcavity and random laser-based barcoding. (a) Schematic of enzyme-responsive hydrogel droplets enclosed in a FP microcavity. An array of such lasers is used for barcoding. (b) Schematic of FP microlaser arrays with random lengths, made by splitting of the liquid on micropillars. The corresponding lasing spectra for different scenarios of splitting of the liquid. (c) Optical image of carbon dot fibers under UV illumination and lasing spectra for three different types of carbon dot fibers. The lasing spectra and the laser-threshold values of different fibers were used for the barcoding. (d) Three random lasers (A, B, and C), each with a different laser threshold. When all pumped with the same power, they emit a different spectrum. These random lasers are printed as ink in different patterns, for example, a linear sequence. Image credits: (a) Reproduced with permission from ref (103). Copyright (2022) Wiley-VCH GmbH. (b) Reproduced with permission from ref (58). Copyright (2019) Wiley-VCH Verlag GmbH & Co. KGaA, Weinheim. (c) Reproduced with permission from ref (78). Copyright (2021) The Royal Society of Chemistry. (d) Reproduced with permission from ref (68). Copyright (2020) American Chemical Society.

There are a few examples of Fabry–Pérot (FP) microcavity lasers being used as barcodes. In one report hydrogel droplet arrays were sandwiched into a thin FP microcavity (Figure 8a).^103^ The laser emission could be tuned by changing the structure of the hydrogel with an enzymatic reaction. At different positions within the array, different swellings could be achieved through different reaction times or enzyme concentrations. By varying the pumping energy, multiple emission states were attained, which enhanced the information-encoding capacity.

The smallest FP microcavities used for barcoding have been cadmium-sulfide (CdS) nanowires (diameter of ∼200 μm and length in the range of 3.5–7 μm).^59−61^ Due to their high refractive index, their smooth end-facets provide high reflectivity, enabling the FP-like operation. Although the *Q*-factor can be low (∼50), this is compensated by a very high gain, so enabling the lasing. The nanowires were pumped with a pulsed laser at 485 nm and had the lasing output at ∼520 nm. Nanowires of different lengths had different lasing spectra and can be used as unique barcodes. The spectrum contained from one up to ∼5 spectral peaks in a relatively narrow spectral region (<10 nm).

In another example, an organic microlaser array with dual lasing emission at 660 nm or/and at 720 nm has been demonstrated for cryptographic primitives.^58^ An array of organic single-crystalline microlasers of random lengths has been assembled. It was produced by trapping a thin layer of a solution between a template of micropillars and the target substrate. Due to the rapid evaporation rate of the solvent, the solution split asymmetrically, as shown in Figure 8b.

The lasing output depended on the length of the microcavity. The barcode was encoded for four different cases of lasing: no lasing, lasing at 660 nm or at 720 nm, and lasing at both (660 and 720 nm) wavelengths (Figure 8b).

### Barcoding Based on Distributed-Feedback Lasers

One example of single-mode laser barcodes is the ultrathin (approximately down to 200 nm), mechanically flexible and substrate-less organic distributed-feedback (DFB) laser.^79^ Here, the cavity consisted of an organic semiconductor layer, deposited onto an undulating polymer grating, produced beforehand by UV nanoimprint lithography. The emission spectrum of such a laser was tuned by changing the period of the diffraction grating. By changing the undulating period, the emission wavelength could be changed in steps of 1 nm. Taking into account the 50 nm-wide gain spectrum of the organic semiconducting polymer, around 50 independent spectral channels could be encoded. This number could be further increased by combining several organic semiconducting polymers with different gain spectra.

Furthermore, liquid-crystal-based, single-mode microlaser barcodes have been demonstrated.^88^ A dye-doped cholesteric liquid crystal (LC) was inkjet-printed onto a microtemplate to produce a laser array. In one of the two inks used, the chiral dopant was photoresponsive, so its isomerization with UV illumination changed the laser’s emission wavelength. In each field of the laser array a bit of information was encoded by either containing the photoresponsive chiral dopant (“1”) or not (“0”).

### Barcoding Based on Random Lasers

A few different barcoding strategies using random lasers have been reported. None of them use the sharp spectral lines present in the lasing spectrum for encoding the barcode, but instead are usually based on the lasing threshold. In one instance, the central lasing wavelength and the threshold were varied by the size of the light-scattering particles.^89^ Carbon dot fibers have also been reported as random lasers for barcoding (Figure 8c).^78^ The central lasing wavelength and the laser-threshold values of the fibers were tuned by varying their diameters and their fabrication parameters.

Since it is difficult to reliably make and read a lot of different laser thresholds, random laser barcodes are frequently used in conjunction with graphical multiplexing. Random lasers are typically a mixture of a dye and scattering particles, so they can be easily applied to different surfaces as inks, using printing techniques to form a graphical pattern. For example, a so-called smart ink was made from a mixture of Rhodamine 6G and Ag nanoparticles embedded in a PVA film (Figure 8d).^68^ The information was encoded by the presence or absence of the Ag particles. With the particles present, random lasing was emitted, while without the particles, there was only spontaneous emission. Furthermore, to encode more information the threshold was varied by changing the concentration of the dye, so that three different thresholds could be distinguished. Three inks (A, B, and C) with different lasing-threshold energies of 26, 35, and 85 mJ/cm^2^, respectively, were used. One way of reading such barcodes is to measure the laser threshold for each area by gradually increasing the power of the pump laser. In the paper, however, they were pumped at a fixed energy density of approximately 35 mJ/cm^2^, which was near the threshold value of ink B. The three inks had emissions of different intensity and spectral width, which was used to read the pattern.

## Applications

### Cell Tagging and Tracking

Cell tagging and tracking are indispensable in many biological research fields, from cancer research to developmental biology and studies of cell differentiation, to name just a few. Here, we will discuss the various reported strategies of tagging cells using optical microcavities and microlasers.

#### In Vitro

**Figure 9:**
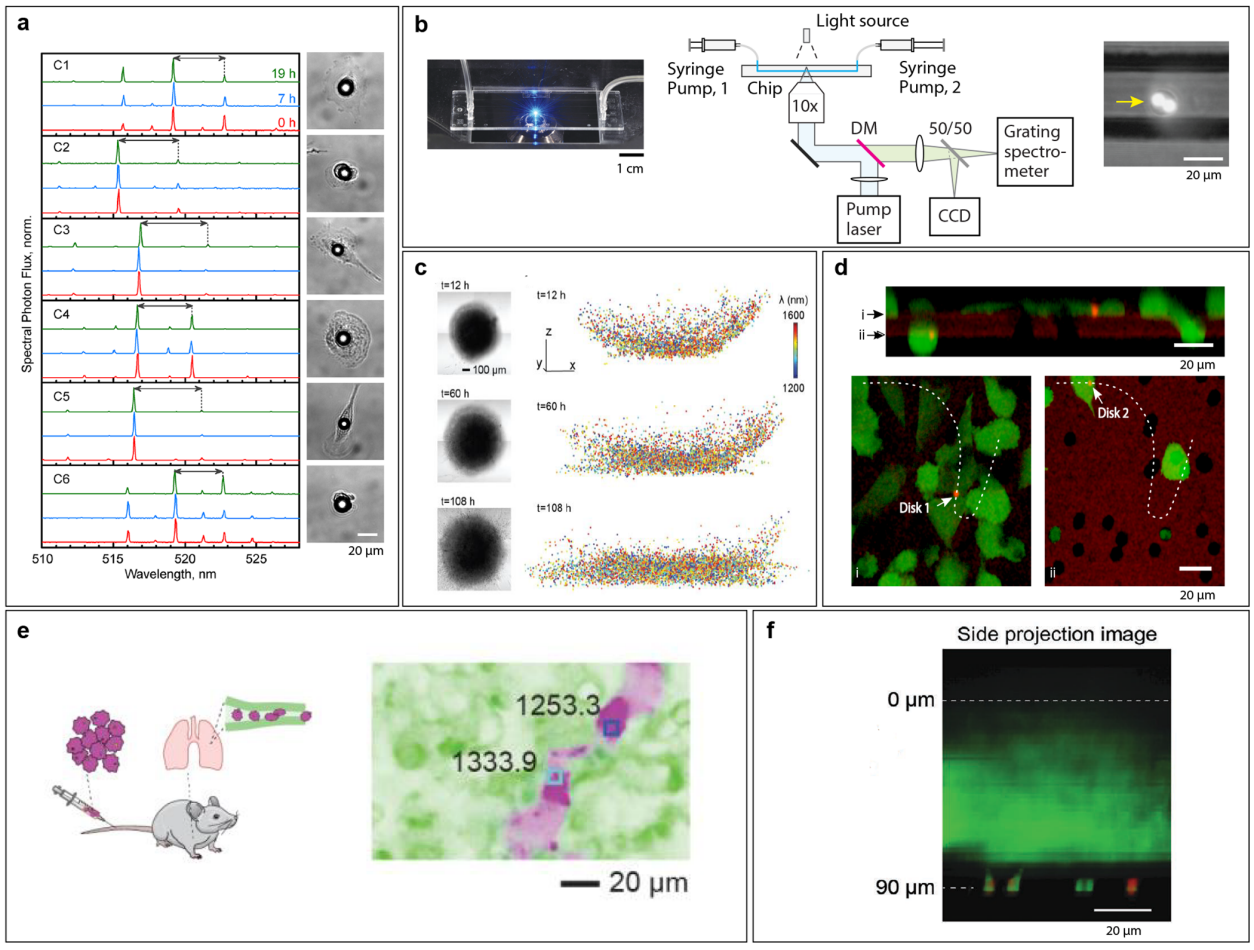
Applications of microcavity-based barcodes for cell tagging and tracking. (a) Spectra of microbead lasers internalized in cells at different time intervals demonstrate their stability and therefore long-term cell tracking. (b) Demonstration of a microfluidics setup used to read spectra from microcavities inside cells. Hundreds of cells were flowed from one side to the other and back. This setup demonstrates the tracking of free-floating cells. (c) Optical transmission images of the tumor spheroid at three different times (left) and the spatial distribution of microdisk lasers inside the spheroid tumor (right). The color represents the lasing spectra. (d) Confocal images of cells containing microdisk lasers (red) passing through holes in a membrane. (e) Cells containing microdisk lasers and stained with a fluorescent dye were injected into the tail vein of a live mouse. After a few minutes, the lung tissue was removed from the mouse for ex vivo imaging to show that microdisks can be used as intracellular tags. (f) Two-photon fluorescence images of subcutaneously injected microbeads into the abdomen of an anesthetized live mouse. Lasing was detected at a depth of ∼90 μm from the surface of the skin. Image credits: (a) Reproduced with permission from ref (95). Copyright (2015) American Chemical Society. (b) Reproduced with permission from ref (96). Copyright (2017) The Royal Society of Chemistry. (c,e) Reproduced with permission from ref (85). Copyright (2019) Springer Nature. (d) Reproduced with permission from ref (57). Copyright (2018) The Author(s), http://creativecommons.org/licenses/by/4.0/. (f) Reproduced with permission from ref (97). Copyright (2021) Wiley-VCH GmbH.

In 2015 an intracellular laser, fully contained within the cell, was demonstrated.^91,95^ Different types of cells, including HeLa, NIH 3T3, and HEK 293, were able to uptake 10–20 μm diameter microbeads via phagocytosis. The cells containing microcavities remained viable for at least 4 weeks. The lasing spectrum of each cell was recorded at different times, making it possible to unambiguously distinguish the cells in time and track their movements (Figure 9a). While pumping the cells well above the threshold for an extended period of time (several thousand pump pulses) could result in cell damage, only one excitation pulse was enough for the complete spectrum to be obtained. Barcoding via fluorescent microspheres was demonstrated in both the spontaneous emission regime and in the lasing regime.^56,91,95^

The cells can naturally uptake multiple microspheres.^56,91,95,96,104^ This enables the efficient tagging of a large number of cells. In one such example, near-infrared intracellular WGM microlaser probes with a narrow emission (approximately 2 nm) were produced from an organic material and coated with a silica shell.^104^ By varying the diameter of the microspheres from 3 to 5 μm, 150 uniquely distinguishable barcodes could be produced. The microcavities were internalized by the cells, which consequently contained up to 5 microcavities. The FSR of each microcavity and the number of microcavities contained in each cell were used as a barcode. By increasing the microsphere-diameter range to 3–10 μm and by using up to 5 microcavities in each cell, the number of unique barcodes could in principle reach 10^12^.

The motility of the cells attached to a substrate or inside a tissue is practically negligible in comparison to free-floating cells, which can completely change their relative positions in a short time. For these, tracking represents a much greater challenge and is sometimes impossible when using conventional methods. The tracking of cells containing polymer WGM microcavities in a dispersion was demonstrated by using a microfluidic channel.^96^ Hundreds of cells were flowed in one direction through the channel, and their spectra were read (Figure 9b). Later, the cells were flowed back and the spectra were read again. A total of 128 cells containing unique barcodes could be traced back to the initial sample.

The polystyrene microbeads used in early demonstrations were relatively large compared to the cells and could have a negative impact on them. In the literature on this subject the internalization of large particles (up to 20 μm) of different materials and shapes by various cells has been studied.^105−110^ Although none of these studies found any significant effect on the cell’s viability and cytotoxicity due to the microparticles when studying the cells for up to 10 days, there is a strong chance that particles of such a size still have a subtle biological influence on the cells. Therefore, smaller microcavities were developed and used for tracking the cells. Quantum-dot-based microbead lasers with single-mode emission were used for in vitro and in vivo imaging.^97^ To check the capability of the microbeads for in vitro cell tracking, murine breast-cancer 4T1 cells were incubated with microbeads ∼4 μm in diameter to induce cellular uptake. A variation in the emission wavelength of ∼0.3 nm was observed over 3 h, probably due to changes in the surrounding refractive index caused by cellular processes or random migration of the microbeads inside the cell. The microbeads were reported not to influence the cell’s viability. Furthermore, the CdS nanowire lasers were also shown to be easily internalized by cells, with no effect on the cells’ viability, making them useful for cell-tagging applications.^59^

To further reduce the size of the intracellular barcodes, microdisk lasers made of semiconductor material (InAlGaAs or InGaAsP) were employed.^85^ The microdisk lasers had a diameter of 2 μm and a thickness of 400 nm. The lasers were also coated with a layer of silica to achieve biocompatibility, as well as to reduce the effect of the external refractive index on the lasing wavelength. After 30 h, a coated microdisk exhibited only a 0.4 nm variation in the lasing wavelength, while an uncoated microdisk showed a variation of 11 nm. To demonstrate the compatibility of a microdisk laser as an intracellular probe, time-lapse imaging was used to monitor the migration and proliferation of MDCK-II cells containing laser particles for several days. Furthermore, for the large-scale cell tracking, a 3D tumor spheroid model of polyclonal 4T1 breast-cancer cells was used, with the cells being tagged by microdisk laser particles (Figure 9c). A single-cell spheroid contained ∼70,000 microdisks. Computer-automated 3D scans were conducted to track the laser particles, and 75–80% of the particles were tracked for a period of about 24 h. After 125 h, only ∼1% of the particles was still tracked. This may be due to the fact that the cell spheroid increases in size and thus the signal cannot be received from beyond a certain thickness. Furthermore, the microdisk lasers emit predominantly in a plane and their orientation inside the cell changes with the cell’s movement, which causes the signal intensity to fluctuate over time and even intermittently lose the signal. This problem has been effectively solved with a recently proposed strategy to incorporate nanoscale light scatterers onto the microlasers,^93^ resulting in an omni-directional emission. To compare, the laser power output varied less than 10 dB when a microdisk with an omni-directional emission changed its orientation, whereas in the case of conventional microdisks the variation was more than 24 dB.

The disk lasers made out of an AlGaInP multi-quantum-well structure were also used for cell tagging.^57^ One of the benefits of using this material, apart from the small size, is the avoidance of arsenic, which is toxic. These lasers also emit in the visible spectrum instead of the infrared, which allows the use of regular cameras, detectors, and spectrometers, which are usually more common and less expensive. On the other hand, if the emission is in the infrared region, the readout of the barcode is completely separated from the light generated by the biological stains, which emit mostly in the visible spectrum. The lasing threshold of these nanodisk lasers was 500-fold below the pulse energy typically used in two-photon microscopy. The nanodisks were efficiently internalized by a number of different cell types, including human macrophage, mouse neuron, human T, and NIH 3T3 cells. Because of their small size, each cell can uptake multiple disk lasers without disturbing the cell processes. The lasing within the cells was demonstrated to be very stable with a wavelength shift of less than 50 pm. An estimated 10^9^ cells could be uniquely tagged by assuming 6 nanodisks per cell. Moreover, these nanodisk lasers can be used to tag individual cells, even while migrating through tiny holes with diameters down to 5 μm, as shown in Figure 9d. This could facilitate the study of cancer invasion, because there the cell migration through small pores and epithelial layer plays an important role.

Similar to the above, cells can be tracked in flow cytometry. Though flow cytometers usually only contain a small number of fluorescence channels, a high-resolution spectrometer can be included to read a high-resolution spectrum from an intracellular microcavity barcode. This makes possible measurements that were not possible before. For example, in the case when the cells are analyzed at two time points, by flowing them twice through the cytometer, it is currently not possible to know how each individual cell changed, only the population averages are measured. Also in conventional flow-cytometry, another limitation is the number of channels that can be simultaneously measured due to the spectral overlap. Recently, a multipass flow-cytometry technique based on barcoding using microdisk lasers was demonstrated to address these issues.^111^ This makes it possible to measure the same cells at different time points, for example, before and after a drug treatment. A larger number of parameters without overlap was also demonstrated, by first measuring one set of parameters, then photobleaching the probes, restaining, and measuring the second set of parameters. Cell sorting in microfluidics based on the intracellular microcavity spectra at a kilohertz rate was also demonstrated.^112^

#### In Tissues and in Vivo

Long-term tracking of a large number of cells in vivo is, in general, extremely challenging. Microcavities are great candidates for in vivo tracking. The positions of sharp emission lines can be easily detected, even from deep tissue, and are relatively unaffected by the tissue scattering, absorption, and autofluorescence. There have been a few reports about barcoding cells in biological tissues by using microlasers, but the real long-term tracking of a large number of cells has not been demonstrated until now.

As a proof of concept, cells containing microdisk lasers and stained with a fluorescent dye were injected into the tail vein of a live mouse (Figure 9e).^85^ After 15 min the lung tissue was removed from the mouse for ex vivo imaging. The injected cells were successfully identified and the lasing signal was measured. However, since the tissue was analyzed ex vivo, only a single time point was available. Quantum-dot-based, single-mode WGM lasers were also employed for in vivo imaging and cell tracking.^97^ The microbeads were injected into the abdomen of an anesthetized live mouse. Lasing was detected at a depth of ∼90 μm from the surface of skin (Figure 9f).

In another example, polymer WGM microcavities were used simultaneously for sensing, the 3D localization of cells and barcoding deep inside tissues.^65^ The microcavities were coated with a polymer, responsive to pH or temperature, which enabled specific sensing. At the same time, the unique spectrum of each microcavity enabled the decomposition of a diffuse signal into contributions from individual microcavities and in this way their localization. The unique spectra could, in this case, also be used for long-term barcoding and the tracking of individual cells through highly scattering tissues.

Furthermore, CdS-based nanowire lasers were reported for the in vivo tracking of cells.^60,61^ A multilayer collagen coating was deposited onto the CdS nanowires using a layer-by-layer assembly method to promote internalization by the macrophage cells. The cell tracking was first performed in vitro, by growing the cells on a hydrogel, so mimicking the tissue. The cells containing the nanowires were then injected into the subretinal layer of the eye. More than 10 transplanted cells were individually tracked for 28 days. The nanowire lasers were simultaneously used as barcodes and as a contrast agent for optical coherence tomography (OCT). Due to their high refractive index they strongly scatter light and therefore provide a strong OCT signal (25 dB enhancement compared to the surrounding retinal layers). OCT provided 3D position information on the nanowires as well as enabling deeper imaging in the tissue (700 μm) compared to the fluorescence alone (400 μm), while at the same time the lasing in visible light provided a unique spectrum from each nanowire, which was used for barcoding. This demonstrated the feasibility of in vivo single-cell migration tracking with the dual-modality imaging system.

### Product Labeling and Anticounterfeiting

A photonic barcode can be used in two main ways for anticounterfeiting. First, a barcode can be made very complex so it is very difficult or even impossible (physical unclonable function) for an individual to reproduce it. Second, a barcode can be made in such a way that it gives the right information only if it is read in a particular way. Optical microcavity barcodes can be used in both of these ways.

**Figure 10:**
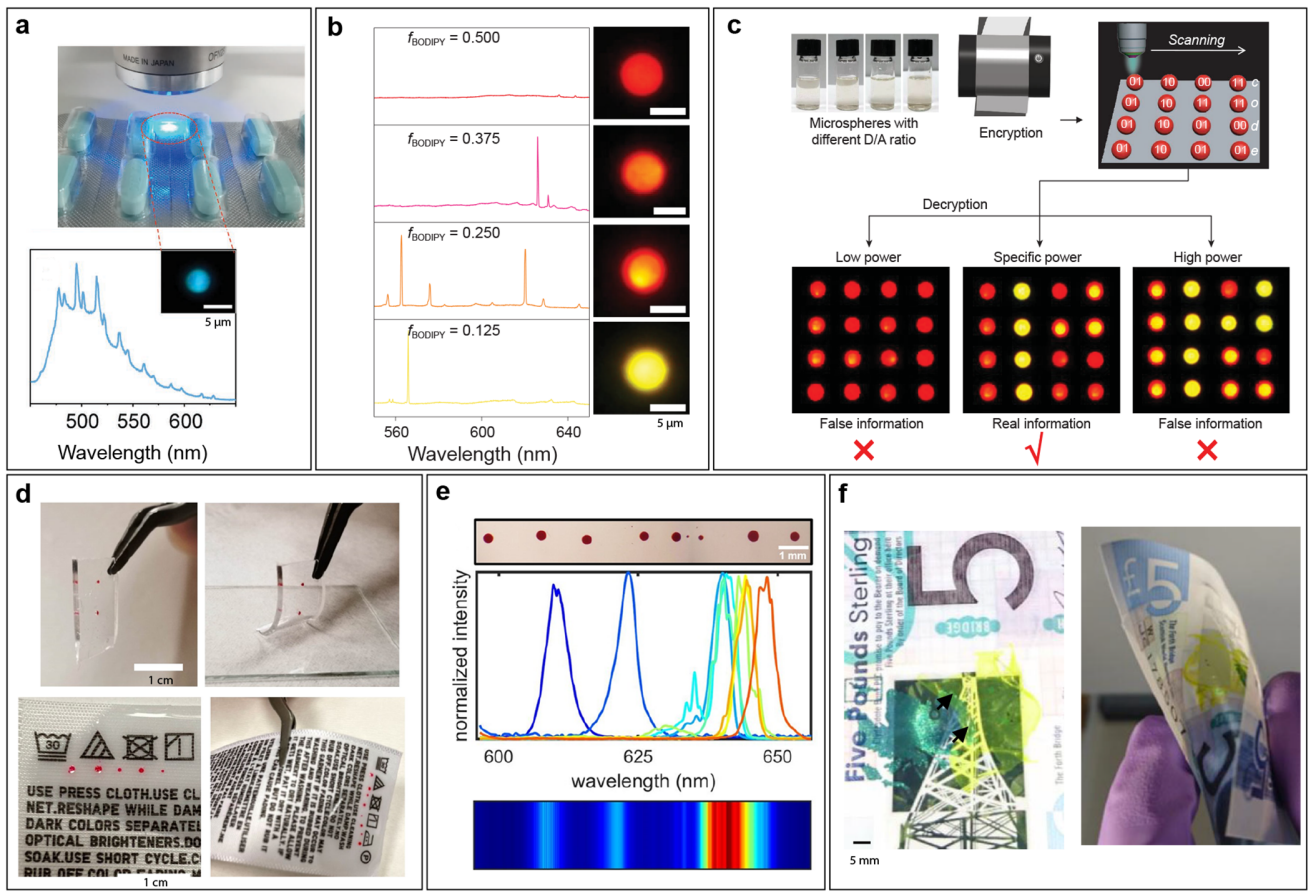
Applications of microcavity-based barcodes for product labeling and anticounterfeiting. (a) Demonstration of a microdisk cavity stamped onto a drug package for anticounterfeiting applications along with its spectrum. (b) WGM spherical lasers with different ratios of acceptor to donor, which results in different lasing at the same pump fluence. (c) Coding by utilizing the four possible lasing states (as shown in panel b), which must be read at the right pumping power to yield the correct information. (d) A label containing droplet microcavities embedded in a polymeric matrix and attached to clothing. (e) Representation of an array of droplets as a photonic barcode and their spectra. (f) A thin, undulating membrane (yellow patch) that acts as a DFB laser is attached to the banknote for anticounterfeiting. The lasing remains stable even when the banknote is bent. Image credits: (a) Reproduced with permission from ref (113). Copyright (2017) Wiley-VCH Verlag GmbH & Co. KGaA, Weinheim. (b,c) Reproduced with permission from ref (114). Copyright (2020) The Author(s). (d,e) Reproduced with permission from ref (115). Copyright (2021) American Chemical Society. (f) Reproduced with permission from ref (79). Copyright (2018) The Author(s).

For example, a photonic barcode for anticounterfeiting was made by depositing dye-doped organic microdisks onto a surface.^113^ The disks were prepared by precipitating polystyrene dissolved in a solvent. The diameter of the microdisk could be varied in the range 2–20 μm by changing the amount of water added to the solution. To show their applicability as a security tag, the microdisks were stamped onto a drug package, as shown in Figure 10a. For anticounterfeiting a dye that changes the emission color under UV irradiation was used in the microdisks. To obtain the correct spectrum from the microdisks, they first have to be illuminated by UV light. Another example of photoswitchable microcavities are the microhemispherical cavities arranged in 2D patterns.^100^ These were prepared from fluorescent photochromic diarylethenes. The emission from a microcavity can be switched by exposure to UV and visible light. The microcavity array performs as a double authentication, first to read the 2D luminescent pattern of the photoswitchable pixels, while another method of authentication is to read the spectrum from each of the microcavities.

The two examples of stimuli-responsive lasing emission described above only have two possible states, which does not make the label particularly safe. Alternatively, four emission states have been achieved by using a donor–acceptor pair in a microsphere laser.^114^ By changing the pump laser fluence, the following cases were achieved: no lasing, lasing at a longer wavelength, lasing at two wavelengths simultaneously and lasing at a shorter wavelength only. The threshold fluence for these states depended on the donor–acceptor ratio; so by varying it, the four states could also be achieved in different microcavities when all were illuminated at a fixed fluence (Figure 10b). To increase the amount of encoded information, microlasers with different donor–acceptor ratios were arranged into 2D arrays (Figure 10c). The correct emission pattern could only be read by applying a predefined excitation fluence. Besides the above-discussed multiple-emission-states strategy, the concept of multidimensional information encryption has also been introduced by realizing thermoresponsive dye-doped cholesteric liquid-crystal microlasers.^116^ To increase the security of the barcode, three parameters were used simultaneously to encode the barcode: the single-mode emission wavelength, the left- or right-handed circular polarization of light and the temperature responsiveness. The lasing wavelength can be chosen by varying the concentration of the chiral dopant. Left or right-handed circular polarization of the emission can be achieved by using a chiral dopant of the corresponding handedness. How much the lasing wavelength is dependent on the temperature is dependent on the particular combination of the liquid crystal and the chiral dopant. Therefore, all three emission parameters are only dependent on the materials used and their concentrations.

A smart photonic label containing liquid droplet microcavities was reported, which can be attached to different kinds of substrates such as paper and fabric (Figure 10d).^115^ The dye-doped droplets with diameters in the range 100–1000 μm were encapsulated into a polydimethylsiloxane (PDMS) matrix. Whispering-gallery-mode lasing as well as random lasing (by the addition of scattering TiO_2_ micropowder) were demonstrated. The lasing was controlled by the size of the droplets as well as the concentration of TiO_2_. Using two types of lasing increases the security, as the barcodes are then more difficult to replicate. The droplets can be arranged in a pattern, enabling the encoding of information (Figure 10e).

The 2D perovskite microlasers (Figure 7d)^102^ and random lasers made out of carbon dot fibers (Figure 8c)^78^ have also been employed for anticounterfeiting. The geometry-dependent lasing threshold and lasing wavelength can be used together for secure information encoding. The already-mentioned membrane DFB lasers can also be used for product labeling and anticounterfeiting. They are produced as a free-standing film, which can contain several different diffraction gratings at different locations—several different lasers. The film can be transferred to a hard surface and the combined spectrum of all the contained lasers can be used as a barcode, for example, for banknote authentication, as shown in Figure 10f. The emission spectrum was shown to be stable for 200 days, as well as when exposed to twisting of the banknote.

## Discussion and Future Prospects

The application of microcavities and microlasers to barcoding is a relatively new area of research; therefore, there are many opportunities in the future in terms of the development of new types of microcavities, optimization of their properties and novel applications. Not only the microcavity barcodes themselves, but also the hardware and algorithms to read them need further development. The advances in compact light sources and detectors, such as on-chip spectrometers, can bring microcavity- and microlaser-based optical barcoding closer to everyday use in research and industry, as well as for consumers.

### New Types of Microcavities and Lasers for Barcoding

WGM microcavities are the main type of microcavities currently in use for barcoding. Other microcavity types, such as nanowire lasers, distributed feedback lasers and random lasers, have been studied less in this regard, hence there are plenty of unexplored opportunities. Distributed-feedback lasers and photonic crystal lasers are promising for barcoding since their lasing wavelength can be easily tuned by changing the period of the structure, which enables the encoding of information. In general, two-dimensional lasers are especially useful for barcoding and anticounterfeiting since they can be easily mass produced, can be applied to almost any surface and are less sensitive to the pumping position and the detection direction.

For some applications such as cell tagging, the barcodes should be as small as possible. There are a number of metallic and plasmonic nanolasers reported,^117−123^ with the smallest having a diameter of only 22 nm.^117^ The emission wavelength of the plasmonic lasers can change, depending on their shape as well as by selecting a different gain material,^117,124^ which would enable barcoding. However, these lasers typically have broader emission peaks and their emission might not be so easily tuned across a very wide wavelength range, which may result in a smaller number of barcodes. The operation of a spaser has also been demonstrated inside a live cell.^117^

Random lasers are also relatively underexplored in terms of barcoding. Their intrinsic random-emission spectrum could be ideal for generating a large number of barcodes as well as for anticounterfeiting. Apart from random lasers, other lasers with a complex cavity geometry can also have a spectrum with a large number of independent peaks. These are, for example, chaotic lasers with various shapes, such as D-shaped cavities, stadium-shaped cavities or just slightly deformed or imperfect WGM cavities.^125−127^ Due to the rich emission spectra, these lasers have great potential for barcoding applications, but have not yet been employed for this purpose. Apart from opportunities, there are also challenges to be overcome. For example, for cavities with a large number of optical modes there will be mode competition and large sensitivity to external influences and pumping. This might change the barcode. On the other hand, the existence of a number of modes can be exploited for anticounterfeiting. Namely, by pumping the laser at a particular position or using a particular light pattern (e.g., a spatial light modulator) only some selected modes can be brought above the threshold.^73,128^ For anticounterfeiting a user could get the right information from the microlaser only by using the right pumping pattern.

### Increase in the Number of Unique Barcodes and the Amount of Encoded Information

A number of options have been demonstrated to increase the number of unique barcodes, such as combining multiple barcodes, arranging them in patterns and using different gain materials. There are, however, numerous other, largely unexplored possibilities for multiplexing. These include other properties of the output from the microcavity. Various emission patterns,^129^ angular momentum^130^ and polarization, including vector beams^131^ have been demonstrated and could be studied in terms of barcoding. Some of these types of multiplexing are used in optical communications, so some of the concepts could be copied from there. With the advent of more complex encoding schemes the algorithms used to interpret the barcodes also have to become smarter. There have been reports of using machine-learning methods to analyze the output of microlasers,^132^ but they have not been applied to barcoding.

The most common way to increase the number of unique barcodes is to combine more microcavities. In most cases these microcavities within a group are separated enough from each other so that there is no influence between them. However, they can also be combined so that the emission of each microcavity is dependent on the optical properties of others, enabling more diverse emission possibilities. For example, WGM lasers each having a different gain medium can be coupled together, so that each microcavity can serve as both a light source and a light modulator for the other microcavities.^133^

Furthermore, encoding useful information into microcavity barcodes is important for their widespread adoption; however, it is still in its infancy. Some concepts have been demonstrated, but are complex and encode a limited amount of information. In this respect scalability is the main problem to be solved. It is possible that 2D lasers such as DFB lasers,^79^ made by scalable technologies such as nanoimprint lithography, could be used to solve these issues and enable controllable encoding of useful amounts of information.

### Novel Applications of Microcavity Barcodes

One of the most promising applications of optical microbarcodes is biomedical research. However, most demonstrations were more a proof of concept. Therefore, a clear future direction is to employ the barcoding to solve some real biological questions both in vitro and in vivo. Some promising applications are emerging such as the multipass flow cytometer for measuring more different parameters of the same cells at two time points, which is being commercialized by the company LASE Innovation Inc. A very important application of microcavities is biosensing, even down to single molecules. By combining barcoding and sensing a truly unique multimodal platform could be realized. A few cases of the multimodal use of microcavities have been demonstrated, combining barcoding with imaging or sensing. There are, however, a number of other possibilities. As an example, due to the absorption of light by the fluorescent materials within microcavities, they could be used for photoacoustic imaging and photothermal therapy. The same particles could also be employed as drug carriers.

Currently, the need for a fluorescence excitation source, filters and a spectrometer makes the use of spectral microcavity barcodes more complex in comparison to standard macroscopic barcodes. For microlaser-based barcodes a pulsed laser is also required for the excitation. However, in the future with the development of miniaturized pulsed lasers and integrated spectrometers their use might become more widespread.

## Conclusions

To conclude, microcavity- and microlaser-based barcodes are a very powerful tool which holds great promise. However, it is only in its infancy. By developing new kinds of microcavities as well as optical systems for their detection, they could become both a tool in research as well as useful for labeling products in everyday life.
